# Serial serum interleukin-8 trajectories are associated with progression from *Helicobacter pylori*–associated precancerous lesions to gastric cancer: a prospective longitudinal cohort study

**DOI:** 10.1186/s13099-026-00876-8

**Published:** 2026-09-23

**Authors:** Atteyat A. Semeya, Raafat S. A. Abdel Hafez, Rasha Elgamal, Abeer Badr, Mahmoud Ibrahim Orabi, Shimaa R. Younis, Mahmoud M. Morgan, Mahmoud Ahmed Hassan, Mohamed Ramzy Alawy, Amira A. A. Othman

**Affiliations:** 1 Hepatology, Gastroenterology and Infectious Diseases Department, Benha Teaching Hospital, Benha, Egypt; 2 Internal Medicine Department, Damanhour Medical National Institute, Damanhour, Egypt; 3 Clinical Pathology Department, Faculty of Medicine, Suez University, Suez, Egypt; 4 Internal Medicine Department, Shebin Elkom Teaching Hospital, Menofia, Egypt; 5 Hepatogastroenterology Department, Faculty of Medicine, Al Azhar University, Assiut, Egypt; 6 Medical Oncology Department, South Egypt Cancer Institute, Assiut University, Assiut, Egypt; 7 Hepatology, Gastroenterology, and Infectious Diseases Department, Faculty of Medicine, Suez University, Suez, Egypt; 8 Gastroenterology Department, El Monira General Hospital, Cairo, Egypt; 9 Internal Medicine Department, Faculty of Medicine, Suez University, Suez, 43511 Egypt

**Keywords:** Interleukin-8, *H. pylori*, Stomach neoplasms, Atrophic Gastritis, Metaplasia

## Abstract

**Background:**

Gastric cancer remains the fourth leading cause of cancer-related mortality worldwide, with delayed diagnosis contributing to poor prognosis. Current biomarkers lack sensitivity for early detection. Interleukin-8 (IL-8), a pro-inflammatory chemokine implicated in H. pylori-associated gastric carcinogenesis, has not been rigorously evaluated in longitudinal studies for its ability to predict malignant progression in patients with precancerous lesions. Aim: To investigate whether serial serum IL-8 measurements predict progression to gastric cancer among Egyptian patients with H. pylori-associated atrophic gastritis and intestinal metaplasia over 24 months of follow-up.

**Methods:**

a prospective, longitudinal biomarker, multicenter cohort study enrolled 200 participants across 5 Egyptian centers: 50 with atrophic gastritis, 50 with intestinal metaplasia, 25 with H. pylori-positive gastric cancer, 25 with H. pylori-negative gastric cancer, and 50 healthy controls. All underwent baseline clinical assessment, laboratory evaluation (including CEA, CA19-9), H. pylori testing (stool antigen and urea breath test per Maastricht VI), and endoscopy with histopathology (Updated Sydney System). Patients with precancerous lesions were followed for 24 months with serial IL-8 measurements at 0, 6, 12, 18, and 24 months, and repeat endoscopy at study completion. Group-based trajectory modeling was performed blinded to outcome status. Multivariable logistic regression with penalization was used due to low event count. Time-dependent ROC analysis used a cumulative/dynamic approach.

**Results:**

Of 100 patients with precancerous lesions, 94 completed follow-up, and 9 progressed to gastric cancer (7 from intestinal metaplasia, 2 from atrophic gastritis). Baseline IL-8 levels demonstrated a progressive increase across groups (controls: 9.7 ± 3.2 pg/mL; atrophic gastritis: 42.1 ± 11.8; intestinal metaplasia: 64.8 ± 14.3; gastric cancer: 107.4 ± 24.6; *p* < 0.001). IL-8 elevation in gastric cancer was independent of H. pylori status across all stages (*p* > 0.50 for all stage-stratified comparisons, though these subgroup analyses are limited by small sample sizes and may be considered exploratory). Baseline IL-8 > 52.3 pg/mL was associated with progression risk, AUC 0.84, sensitivity 88.9%, specificity 78.0%, and negative predictive value 98.5%, significantly outperforming CEA (AUC 0.62) and CA19-9 (AUC 0.58). Time-dependent AUC increased to 0.91 at 12 months and 0.94 at 18 months. Trajectory modeling, performed blinded to outcome, identified three patterns: Stable-Low (*n* = 42), Moderate-Rising (*n* = 49), and High-Accelerating (*n* = 9). The acceleration point in the High-Accelerating group preceded clinical cancer diagnosis by a median of 6 months (range 3–12), however, this exploratory finding requires external validation. In multivariable analysis using Firth penalized regression, baseline IL-8 (aOR per 10 pg/mL: 2.31, 95% CI: 1.48–3.61, *p* < 0.001) and intestinal metaplasia (aOR: 4.22, 95% CI: 1.31–13.61, *p* = 0.016) were independently associated with progression.

**Conclusions:**

In this prospective longitudinal biomarker study, serial serum IL-8 trajectories were significantly associated with progression from H. pylori–associated precancerous lesions to gastric cancer. Rising IL-8 levels preceded clinical diagnosis by approximately 6–12 months, suggesting potential utility for risk stratification. A Baseline IL-8 > 52.3 pg/mL was independently associated with progression (adjusted HR: 6.94, *p* < 0.001), but this exploratory trajectory is hypothesis-generating and requires external validation before causal interpretation. However, these findings are exploratory and hypothesis-generating.

## Introduction

Gastric cancer remains a significant global health burden, ranking as the fifth most commonly diagnosed malignancy and the fourth leading cause of cancer-related mortality worldwide. According to the GLOBOCAN 2020 estimates, there were approximately 1.09 million new cases and 769,000 deaths from gastric cancer globally in 2020 [1]. The disease burden is disproportionately distributed, with over 70% of cases occurring in developing countries, particularly in Eastern Asia, Eastern Europe, and Latin America [2]. Despite advances in diagnostic modalities and therapeutic interventions, the 5-year survival rate remains below 30% in most regions, with global data from the CONCORD-3 program demonstrating that gastric cancer survival has seen only modest improvement over the past two decades [3]. This poor prognosis is primarily attributable to late-stage diagnosis, as early gastric cancer is frequently asymptomatic, and population-based screening programs are not universally implemented [4].

*Helicobacter pylori* infection represents the single strongest risk factor for gastric cancer, classified as a Group 1 carcinogen by the International Agency for Research on Cancer since 1994 [5]. The bacterium colonizes the gastric mucosa of approximately 4.4 billion individuals worldwide, based on 2015 estimates, with prevalence rates exceeding 50% in many developing nations [6]. The carcinogenic cascade initiated by *H. pylori* follows a well-characterized sequence: chronic active gastritis progresses to atrophic gastritis, then to intestinal metaplasia, and ultimately to dysplasia and invasive carcinoma, a process that may span decades [7]. This protracted precancerous phase offers a critical window of opportunity for intervention and early detection, provided that reliable biomarkers exist to identify individuals at imminent risk of progression. The primary outcome of interest in this context, progression to gastric cancer, is defined as histopathologically confirmed invasive adenocarcinoma arising in patients with baseline precancerous lesions.

In Egypt, the burden of *H. pylori* infection and gastric cancer is substantial. A multicenter Egyptian study reported an overall *H. pylori* prevalence of 52% in the Delta region, with higher rates in rural populations and among individuals of lower socioeconomic status [8]. Gastric cancer ranks among the top ten cancers in Egyptian males, with an age-standardized incidence rate of 6.8 per 100,000 according to the national population-based cancer registry program [9]. Despite this burden, organized screening programs are lacking, most patients present with advanced disease, and rising antibiotic resistance has reduced conventional triple therapy efficacy, necessitating optimized regimens [10]. These realities underscore the urgent need for cost-effective, non-invasive biomarkers capable of detecting precancerous progression, yet none have been validated in Egyptian populations.

Current risk stratification relies on endoscopic biopsies, which are invasive, resource-intensive, and impractical for population screening [11]. Serum biomarkers CEA and CA19-9 have inadequate sensitivity/specificity for early-stage disease (diagnostic accuracy < 70%) and lack disease specificity, as they are elevated in colorectal and pancreatic cancers. No established “gold standard” serum biomarker exists for gastric cancer risk stratification; thus, CEA and CA19-9 remain the most widely used comparators despite their limitations [12]. Pepsinogen testing assesses gastric atrophy but fails to capture dynamic inflammation and offers limited predictive value for individual progression risk [13]. These limitations highlight a critical gap: the absence of a non-invasive, biologically relevant tool capable of dynamically tracking carcinogenic progression and identifying patients crossing from benign to malignant transformation.

Interleukin-8 (IL-8) has emerged as a biologically plausible candidate biomarker for *H. pylori*-associated gastric carcinogenesis. IL-8 is secreted by gastric epithelial cells in response to *H. pylori* infection, recruiting inflammatory cells to the gastric mucosa [14]. The sustained IL-8-driven inflammatory response creates a mutagenic microenvironment characterized by reactive oxygen species, DNA damage, and genomic instability [15]. *H. pylori* virulence factors, particularly CagA, directly upregulate IL-8 through NF-κB activation, establishing a mechanistic link between bacterial infection, inflammation, and carcinogenesis [16]. IL-8 is a biologically plausible but non-specific inflammatory chemokine that may provide prognostic information in the context of *H. pylori*-associated gastric carcinogenesis. IL-8 is upregulated by H. pylori through NF-κB activation [16], positioning it as a potentially informative marker of the host inflammatory response to infection. Furthermore, IL-8’s dual role as a neutrophil chemoattractant and promoter of angiogenesis and epithelial-mesenchymal transition [17] makes it a more comprehensive biomarker than markers that reflect only downstream or aggregated inflammatory effects.

Beyond its pro-inflammatory role, IL-8 directly promotes angiogenesis, tumor cell proliferation, and epithelial-mesenchymal transition [17]. In human gastric carcinomas, IL-8 expression correlates with microvessel density and poor prognosis [18], and experimental models confirm that IL-8 transfection enhances tumor growth while IL-8 inhibition suppresses angiogenesis [19]. Cross-sectional studies demonstrate elevated serum IL-8 in gastric cancer patients [20, 21] with diagnostic accuracy of 78% sensitivity and 82% specificity, superior to conventional markers [22]. Although circulating IL-8 represents systemic spillover rather than full intratumoral concentrations, the consistent association between serum IL-8 and adverse outcomes across malignancies supports its utility as a clinically informative biomarker [17]. However, cross-sectional designs cannot establish whether IL-8 elevation precedes malignant transformation. Longitudinal studies examining IL-8 dynamics during gastric carcinogenesis are exceptionally rare [23], and the few available are limited by single-time-point measurements or small populations. Consequently, the natural history of IL-8 across the precancerous cascade remains undefined, and evidence-based thresholds for clinical decision-making are lacking, a barrier to clinical implementation. This gap is particularly pressing in resource-limited settings like Egypt, where endoscopy-based surveillance is impractical, and biomarkers must be both accurate and affordable.

Despite extensive evidence linking IL-8 to gastric carcinogenesis in cross-sectional studies, longitudinal data evaluating whether serial IL-8 dynamics precede and are independently associated with progression from precancerous lesions to invasive gastric cancer remain limited. In particular, the temporal behavior of IL-8 before malignant transformation has not been well characterized in prospective cohorts.

We hypothesized that: (1) serum IL-8 increases progressively across the histological spectrum from normal mucosa to gastric cancer; (2) baseline IL-8 is independently associated with subsequent malignant progression in patients with precancerous lesions; (3) longitudinal IL-8 trajectories distinguish progressors from non-progressors, with accelerating patterns signaling imminent transformation; and (4) IL-8 demonstrates superior prognostic performance compared to conventional, non-specific biomarkers CEA and CA19-9. Therefore, this study aimed to evaluate the longitudinal trajectories of serum IL-8 in patients with *H. pylori*–associated precancerous gastric lesions and to determine whether baseline levels and temporal changes are independently associated with progression to gastric cancer. Secondary objectives included establishing optimal risk stratification cut-off values, characterizing distinct IL-8 trajectory patterns, comparing IL-8 performance with conventional biomarkers, and developing a risk prediction model incorporating clinical, demographic, and biomarker variables.

The clinical significance of this investigation is substantial. If validated, serial IL-8 monitoring could transform current surveillance paradigms by enabling non-invasive, dynamic risk stratification. Patients with low-risk IL-8 trajectories could be safely managed with extended surveillance intervals, reducing endoscopic procedures. Conversely, individuals exhibiting high-risk trajectories would be prioritized for intensive monitoring and early intervention, potentially detecting cancer at curable stages. In the Egyptian context, where endoscopic resources are limited and gastric cancer presents late, such a stratified approach could have profound public health implications. Furthermore, identification of an “IL-8 high-accelerating” phenotype may define a subgroup of patients who would derive particular benefit from enhanced anti-inflammatory strategies or chemoprevention, opening new avenues for personalized risk stratification.

By leveraging a multicenter prospective design with standardized protocols, rigorous histopathological confirmation, and comprehensive longitudinal follow-up, this study aims to generate the first evidence-based framework for incorporating dynamic IL-8 monitoring into gastric cancer surveillance, addressing a critical unmet need in the global effort to reduce gastric cancer mortality.

## Subjects and methods

### Study population and design

This was a prospective, multicenter, longitudinal cohort study designed to evaluate the predictive value of serial serum interleukin-8 (IL-8) measurements for gastric cancer progression in *H. pylori*-infected Egyptian patients with precancerous lesions.

The study was conducted across five high-volume Egyptian gastroenterology and hepatology centers: Banha Teaching Hospital, Shebin Elkom Teaching Hospital, Damnhour Teaching Hospital, Assuit Liver Center, and Suez University Faculty of Medicine. Patient enrollment occurred between January 2022 and January 2024, with a 24-month follow-up period extending to January 2026. The overall study design and management of *H. pylori* infection were guided by the Maastricht VI/Florence consensus [13], while the biopsy protocol and histopathological classification were based on the Updated Sydney System [7].

The study was designed with two complementary components: (1) a cross-sectional comparison of serum IL-8 levels across the full histological spectrum of gastric carcinogenesis, including *H. pylori*-positive and *H. pylori*-negative gastric cancer, to establish the relationship between IL-8 and disease stage independent of infection status; and (2) a 24-month prospective longitudinal cohort study of patients with *H. pylori*-associated precancerous lesions (atrophic gastritis and intestinal metaplasia) to evaluate the predictive value of baseline and serial IL-8 measurements for progression to gastric cancer. Thus, the present investigation constitutes a hybrid design incorporating both cross-sectional and prospective longitudinal components.

All 200 participants underwent standardized upper gastrointestinal endoscopy at baseline. Systematic gastric biopsies were obtained according to the Updated Sydney System and were evaluated by experienced gastrointestinal pathologists for histopathological classification. This assessment was used to confirm normal mucosa (healthy controls), atrophic gastritis, intestinal metaplasia, or gastric adenocarcinoma. In addition, all participants underwent monoclonal stool antigen testing and a 13 C-urea breath test to evaluate active *H. pylori* infection. Infection status was defined by concordant results of the two non-invasive tests; in cases of discordance, histopathological identification of *H. pylori* organisms in biopsy specimens determined the final classification.

Sample size calculation was performed based on the primary outcome of progression to gastric cancer among patients with precancerous lesions. Assuming a 10% progression rate over 2 years in the intestinal metaplasia group based on previous cohort studies [23], and aiming to detect a hazard ratio of 3.0 for progression associated with elevated baseline IL-8 (≥ 50 pg/mL) with 80% power and a two-sided α of 0.05, a minimum of 44 patients per precancerous group was required. To account for an anticipated 15% dropout rate during the 24-month follow-up and to ensure robust subgroup analyses, we enrolled 50 patients each in the atrophic gastritis and intestinal metaplasia groups. The gastric cancer groups (*H. pylori*-positive and *H. pylori*-negative) were each set at 25 patients to allow comparative analyses of IL-8 levels across disease stages, while acknowledging that longitudinal follow-up did not apply to these groups. The healthy control group (*n* = 50) was sized to establish reference ranges and ensure adequate power for cross-sectional comparisons. The final sample of 200 participants exceeded the minimum required for all planned analyses, providing 90% power for detecting clinically meaningful differences in IL-8 trajectories between progressors and non-progressors.

Group allocation was based on baseline infection status and remained fixed throughout all longitudinal analyses, irrespective of eradication outcome. The IL-8-based risk stratification algorithm was developed as an exploratory research framework for patients with histologically confirmed *H. pylori*-associated precancerous gastric lesions (atrophic gastritis or intestinal metaplasia) and was not intended as a population screening tool or a substitute for established surveillance guidelines. Thus, Groups I and II are referred to as *H. pylori*-positive, based on their status at enrollment, noting that they subsequently received eradication therapy per protocol. Group labels reflect baseline status for consistency with original group allocation. (see Table 9).

### Ethical considerations

The study protocol was approved by the Research Ethics Committee of the General Organization of Teaching Hospitals and Institutes, Egypt (Approval #: HB-000130), and conducted in accordance with the Declaration of Helsinki. The protocol was finalized before participant enrollment. As this was a prospective observational cohort study without investigator-assigned interventions, prospective clinical trial registration was not required, consistent with the journal’s submission guidelines. The protocol is maintained as an institutional ethics document and is available for editorial review upon request. The primary study objectives, eligibility criteria, biomarker measurements, follow-up schedule, primary endpoint, and conventional comparative statistical analyses were prespecified in the IRB-approved protocol. Additional analyses, including group-based trajectory modeling, ROC-derived IL-8 thresholds, Kaplan–Meier analyses based on derived cutoffs, decision curve analysis, and development of the IL-8-based risk stratification framework, were performed after completion of the primary analyses as exploratory investigations and are identified as such throughout the manuscript.

All participating centers provided local institutional approval. Written informed consent was obtained from all participants after a detailed explanation of study procedures, including the nature of endoscopic surveillance, blood sampling schedule, and potential risks and benefits. Endoscopic evaluation in healthy controls was performed to ensure histologically confirmed normal gastric mucosa as a reference standard for biomarker comparisons. All procedures were conducted with appropriate ethical safeguards, and the inclusion of healthy controls was approved by the ethics committee as essential for establishing valid reference ranges. Participants were assured of their right to withdraw at any time without affecting their clinical care. Patient data were anonymized using unique study identifiers, and all records were stored securely in compliance with Egyptian data protection regulations.

### Eligibility criteria

Eligible participants were adults aged 18 years or older of both sexes who met group-specific criteria. For Group I (atrophic gastritis), inclusion required endoscopically and histologically confirmed chronic atrophic gastritis with positive *H. pylori* stool antigen testing and no prior history of gastric cancer. Group II (intestinal metaplasia) comprised patients with endoscopically and histologically confirmed intestinal metaplasia, positive *H. pylori* stool antigen testing, and no previous gastric cancer diagnosis. Group III (gastric cancer, *H. pylori*-positive) included patients with incident, treatment-naïve gastric adenocarcinoma confirmed by histopathology and positive *H. pylori* stool antigen testing. Group IV (gastric cancer, *H. pylori*-negative) comprised patients with treatment-naïve gastric adenocarcinoma confirmed by histopathology and negative *H. pylori* stool antigen testing. Group V (healthy controls) included asymptomatic volunteers recruited from the community with normal endoscopic findings, normal gastric histopathology, negative *H. pylori* stool antigen testing, and no history of gastrointestinal disease.

Exclusion criteria were rigorously applied to minimize confounding variables that could influence IL-8 levels independent of gastric carcinogenesis. All blood samples were collected after an overnight fast, between 8:00 and 10:00 AM to minimize circadian variation, and were processed within two hours of collection.

Patients were excluded if they had a history of gastric surgery or gastric malignancy, had received treatment for *H. pylori* infection within six months before enrollment, or had used proton pump inhibitors, bismuth compounds, or antibiotics within four weeks before baseline endoscopy. Individuals with active non-gastrointestinal malignancy or history of any malignancy within five years were excluded, as were those with chronic inflammatory conditions such as rheumatoid arthritis, inflammatory bowel disease, or systemic lupus erythematosus that could independently elevate IL-8. Severe comorbidities, including end-stage renal disease, decompensated cirrhosis, or advanced heart failure precluding endoscopic surveillance, constituted exclusion criteria, as did pregnancy, lactation, or refusal to provide informed consent. The four-week washout period for acid-suppressing medications was mandated because proton pump inhibitors can alter *H. pylori* density and gastric inflammation, potentially affecting both histopathological grading and IL-8 levels [14]. Patients with chronic inflammatory conditions were excluded because systemic inflammation could elevate circulating IL-8 independently of gastric pathology, introducing bias [17].

Additional exclusion criteria were applied to minimize confounding of IL-8 measurements: (1) acute or chronic infection within the preceding 4 weeks, including upper respiratory tract infection, urinary tract infection, or any febrile illness; (2) body mass index ≥ 35 kg/m², given the association between obesity and elevated inflammatory markers; (3) regular use of non-steroidal anti-inflammatory drugs or corticosteroids within 4 weeks before enrollment; (4) diagnosis of metabolic syndrome as defined by International Diabetes Federation criteria; and (5) any condition associated with systemic inflammation not already specified, including uncontrolled diabetes mellitus (HbA1c > 8%) or chronic kidney disease (eGFR < 45 L/min/1.73 m²).

Patients with autoimmune cytopenia (e.g., immune thrombocytopenia, autoimmune hemolytic anemia) at baseline were excluded, as these conditions could independently influence inflammatory markers and potentially confound IL-8 measurements. Additionally, patients receiving any experimental therapy or participating in another clinical trial within 30 days before enrollment were excluded to avoid potential confounding effects on biomarker levels.

### Baseline clinical assessment

All participants underwent a comprehensive baseline evaluation following standardized case report forms. Demographic characteristics, including age, sex, and body mass index, were recorded, along with detailed medical history encompassing comorbidities such as diabetes mellitus and hypertension. Gastrointestinal symptoms were systematically documented using the validated Gastrointestinal Symptom Rating Scale, with particular attention to abdominal pain, bloating, nausea, early satiety, and weight loss. Risk factors for gastric cancer, including smoking status, dietary habits, and family history of gastric malignancy, were also recorded. Vital signs and concurrent medications were documented to identify potential confounders.

### Diagnostic confirmation of *H. pylori* infection

Active *H. pylori* infection was determined using a dual non-invasive testing strategy in accordance with Maastricht VI guidelines to ensure diagnostic accuracy and distinguish active infection from prior exposure [13]. All participants underwent monoclonal stool antigen testing (Amplified IDEIA Hp StAR, Dako, Glostrup, Denmark) and a 13 C-urea breath test. Infection status was defined by concordant positivity on both non-invasive tests. In cases of discordance, histopathological identification of *H. pylori* organisms in endoscopic biopsy specimens, supplemented by rapid urease testing, served as the reference standard for final classification [24].

### Endoscopic procedures and biopsy protocol

All participants underwent upper gastrointestinal endoscopy performed by board-certified gastroenterologists using high-definition video endoscopes (Olympus CV-190, Tokyo, Japan) under conscious sedation with midazolam. The endoscopic examination followed standardized protocols, with careful documentation of findings including the presence of atrophic gastritis, intestinal metaplasia, erosions, ulcers, polyps, or mass lesions.

Biopsy sampling adhered strictly to the Updated Sydney System recommendations [7]. From each patient, two biopsy specimens were obtained from the antrum within 2–3 centimeters of the pylorus, two from the corpus at the mid-portion of the greater curvature, and one from the incisura angularis. Additionally, any visible lesions suspicious for malignancy were biopsied separately. For patients with suspected gastric cancer, additional biopsies were obtained to ensure adequate tissue for histopathological diagnosis and subtyping. All biopsy specimens were immediately placed in 10% neutral buffered formalin for histopathological processing.

### Histopathological evaluation

Formalin-fixed biopsy specimens were embedded in paraffin, sectioned, and stained with hematoxylin and eosin for routine histopathological examination. Modified Giemsa staining was performed for *H. pylori* detection. Histopathological evaluation was conducted independently by two experienced gastrointestinal pathologists who were blinded to clinical data and IL-8 results. In cases of disagreement, consensus was reached through joint review.

For patients with non-malignant findings, gastritis was classified according to the Updated Sydney System, which evaluates the severity of inflammation (mononuclear cell infiltration), activity (neutrophilic infiltration), atrophy (loss of glandular tissue), intestinal metaplasia (presence of goblet cells), and *H. pylori* density, each graded on a visual analog scale from 0 to 3 [7]. Chronic atrophic gastritis was defined by the presence of glandular loss with or without metaplasia. Intestinal metaplasia was subclassified as complete (type I) or incomplete (types II and III) when sufficient tissue was available.

Gastric cancer diagnosis was established exclusively by histopathological examination of endoscopic biopsy specimens. For patients with gastric cancer, tumors were classified according to the Lauren classification as intestinal type, diffuse type, or mixed type. Histological grade was recorded, and staging was performed according to the American Joint Committee on Cancer (AJCC) 8th edition guidelines based on endoscopic ultrasound and cross-sectional imaging when available [25].

Baseline biopsies from all patients were systematically reviewed to exclude the presence of dysplasia or occult malignancy; only patients with confirmed benign precancerous lesions (atrophic gastritis or intestinal metaplasia without dysplasia) were included in the longitudinal cohort. All histopathological assessments for both baseline and follow-up biopsies were performed by two pathologists blinded to clinical data and IL-8 results, with consensus required for diagnosis of dysplasia or malignancy.

### Laboratory investigations

Peripheral blood samples were collected from all participants after an overnight fast. All blood samples were collected after an overnight fast, between 8:00 and 10:00 AM to minimize circadian variation, and were processed within two hours of collection. For complete blood count, 2.5 milliliters of venous blood were collected in ethylenediaminetetraacetic acid tubes and analyzed using automated hematology analyzers (Sysmex XN series) at each participating center. For serum analyses, an additional 5 milliliters of blood were collected without anticoagulant, allowed to clot at room temperature for 30 min, and centrifuged at 1250 × g for 10 min. Serum was aliquoted and stored at -80 °C until analysis.

Liver function tests, including alanine aminotransferase, aspartate aminotransferase, and albumin, as well as kidney function tests, including serum creatinine and urea, were performed using automated chemistry analyzers (Cobas c111, Roche Diagnostics) at each center’s clinical laboratory. Estimated glomerular filtration rate was calculated using the Chronic Kidney Disease Epidemiology Collaboration equation [26].

Conventional tumor markers, including carcinoembryonic antigen and carbohydrate antigen 19 − 9, were measured using electrochemiluminescence immunoassays (Cobas e601 analyzer, Roche Diagnostics) following manufacturer protocols. Reference ranges were established as < 5 nanograms per milliliter for carcinoembryonic antigen and < 37 units per milliliter for carbohydrate antigen 19 − 9.

### IL-8 measurement

Serum interleukin-8 concentrations were quantified using a high-sensitivity enzyme-linked immunosorbent assay (Human IL-8/CXCL8 Quantikine ELISA Kit, R&D Systems, Minneapolis, MN, USA; Catalog # D8000C) performed strictly according to the manufacturer’s instructions. This assay has a minimum detectable dose of 3.5 picograms per milliliter, with intra-assay precision of 4.1 to 6.2% and inter-assay precision of 6.8 to 8.1% as reported by the manufacturer. All samples were assayed in duplicate, and mean values were used for statistical analysis. Laboratory personnel performing IL-8 assays were completely blinded to clinical, endoscopic, and histopathological data. To minimize batch effects, samples from each patient across all time points were analyzed on the same plate whenever possible. Quality control samples with known IL-8 concentrations provided by the manufacturer were included in each assay run to ensure consistency and validity of results.

### Longitudinal follow-up protocol

Patients with atrophic gastritis and intestinal metaplasia (Groups I and II) were followed prospectively for 24 months with serial assessments at baseline, 6, 12, 18, and 24 months. At each follow-up visit, venous blood samples were collected for IL-8 measurement using identical procedures as at baseline. Samples were processed within two hours of collection, centrifuged, and serum aliquots were stored at -80 °C until analysis. Gastrointestinal symptoms were reassessed at each visit using the Gastrointestinal Symptom Rating Scale. Patients were contacted monthly by telephone between scheduled visits to maintain follow-up, document any intercurrent illnesses or medication use, and encourage adherence to the study protocol.

All patients underwent protocol-mandated surveillance upper gastrointestinal endoscopy with biopsy at 12 months (± 4 weeks) in addition to the scheduled 24-month endoscopy. The 12-month endoscopy followed an identical biopsy protocol to the baseline examination: two biopsies from the antrum, two from the corpus, and one from the incisura angularis, with additional biopsies of any visible lesions. Biopsy specimens were processed and evaluated identically to baseline specimens by the same two blinded pathologists, with consensus review for any discrepant interpretations. This 12-month surveillance endoscopy served three critical purposes: (1) to exclude the development of interval cancers that might have arisen before the 24-month time point; (2) to provide histopathological correlation for the 12-month IL-8 measurement; and (3) to enable precise determination of the temporal relationship between IL-8 trajectory acceleration and the appearance of endoscopically detectable neoplasia.

At the 24-month visit, all patients in Groups I and II underwent repeat upper gastrointestinal endoscopy with biopsy following the same protocol as at baseline. Biopsy specimens were obtained from the antrum, corpus, and incisura angularis, as well as from any visible lesions, and were processed and evaluated identically to baseline specimens. For patients who developed alarm symptoms such as dysphagia, unexplained weight loss, gastrointestinal bleeding, or persistent vomiting during follow-up, or those who demonstrated a rapid and sustained increase in IL-8 levels exceeding 50% above baseline, early repeat endoscopy was performed at the discretion of the treating physician.

In accordance with Maastricht VI guidelines recommending *H. pylori* eradication in patients with chronic gastritis, all patients in Groups I and II were offered standardized eradication therapy following baseline endoscopy. The regimen consisted of vonoprazan 20 mg twice daily, amoxicillin 1 g twice daily, and clarithromycin 500 mg twice daily for 14 days, with metronidazole substitution for penicillin-allergic patients. Eradication was confirmed by a negative 13 C-urea breath test at least 4 weeks after treatment completion. Patients with persistent infection received second-line therapy per local protocols.

### Definitions and outcome measures

The following definitions were established a priori and applied consistently throughout the study:

#### Histopathological definitions

*Atrophic gastritis* was defined according to the Updated Sydney System as loss of appropriate glands in the gastric mucosa, with or without metaplasia, graded semiquantitatively from 0 (absent) to 3 (severe) based on the proportion of glandular loss [7]. Only patients with a minimum atrophy grade of 1 in at least one biopsy site were included in Group I.

*Intestinal metaplasia* was defined as the replacement of gastric foveolar epithelium by intestinal-type epithelium containing goblet cells, with or without Paneth cells and absorptive cells. Subclassification was performed morphologically: complete (type I) intestinal metaplasia was characterized by mature goblet cells interspersed with absorptive cells exhibiting a well-defined brush border; incomplete (type III) intestinal metaplasia was characterized by columnar cells with foamy cytoplasm and irregular goblet cells lacking a well-defined brush border [7]. Patients with any degree of intestinal metaplasia in any biopsy site were eligible for Group II.

*Gastric adenocarcinoma* was defined as an invasive malignant epithelial neoplasm of the stomach with glandular differentiation (intestinal type) or consisting of poorly cohesive cells with or without signet-ring morphology (diffuse type), classified according to Lauren criteria [25]. Staging followed the AJCC 8th edition guidelines based on endoscopic ultrasound and cross-sectional imaging when available [25].

### *H. pylori* infection status definition

Active *H. pylori* infection was defined by concordant positive results on both monoclonal stool antigen testing and 13 C-urea breath test, per Maastricht VI guidelines [13]. In cases of discordance between non-invasive tests, histopathological demonstration of *H. pylori* organisms on modified Giemsa-stained biopsy sections with associated gastritis was required for classification as positive. Patients with negative results on all tests were classified as negative.

#### Conventional tumor marker thresholds

Elevated carcinoembryonic antigen was defined as > 5 ng/mL, and elevated carbohydrate antigen 19 − 9 as > 37 U/mL, per standard clinical laboratory reference ranges at all participating centers.

#### Post hoc definition

Based on trajectory analysis findings, a high-risk IL-8 phenotype was defined after trajectory identification but before final outcome ascertainment as: (1) baseline IL-8 exceeding the optimal cut-off (52.3 pg/mL) AND (2) 12-month IL-8 exceeding 64 pg/mL, OR (3) ≥ 50% increase in IL-8 from baseline to 12 months. This exploratory definition was developed to facilitate clinical risk stratification and requires validation in independent cohorts before widespread clinical application.

#### Outcome measures

Based on these standardized definitions, the following outcome measures were prospectively defined to evaluate the clinical utility of IL-8 measurement.

The primary outcome of the study was progression to gastric cancer, defined as histopathological confirmation of invasive gastric adenocarcinoma on follow-up endoscopy biopsy in patients with baseline precancerous lesions, including atrophic gastritis or intestinal metaplasia. This diagnosis required consensus agreement between two independent gastrointestinal pathologists who were blinded to all clinical and IL-8 data. Time-to-progression was defined as the interval between baseline assessment and histologically confirmed diagnosis of gastric cancer. Participants without progression were censored at the time of the last follow-up endoscopy. It is noteworthy that no cases of isolated low-grade or high-grade dysplasia were observed in this cohort. All progression events (*n* = 9) were invasive gastric adenocarcinoma as the first detected neoplastic outcome. Dysplastic changes, when present, occurred only adjacent to invasive carcinoma and were not analyzed as separate outcomes.

Several secondary outcomes were prospectively defined to comprehensively evaluate the clinical utility of IL-8 measurement. The first was the identification of distinct longitudinal IL-8 trajectory patterns over the 24-month follow-up period and their association with subsequent cancer progression, which would enable dynamic risk assessment rather than reliance on a single time-point measurement. The second was the assessment of predictive performance of baseline IL-8, including its sensitivity, specificity, and predictive values for identifying patients who would progress to gastric cancer within the study period. The third secondary outcome was the determination of optimal IL-8 threshold values for clinical risk stratification, specifically identifying cut-off points that best discriminate between patients who will progress and those who will not.

Additional secondary outcomes included a head-to-head comparison of IL-8 predictive performance with conventional tumor markers, namely carcinoembryonic antigen and carbohydrate antigen 19 − 9, to determine whether IL-8 offers incremental value over existing biomarkers. The time-dependent predictive value of serial IL-8 measurements was also evaluated to understand how predictive accuracy evolves as patients are followed over time and whether later measurements provide superior risk stratification compared to baseline assessment alone. Finally, a multivariable risk prediction model was developed incorporating IL-8 along with clinical and histopathological variables, including age, sex, smoking status, and histopathological category, to estimate individual patient risk of progression and to identify independent predictors of malignant transformation.

### Data quality assurance and completeness

Building on this rigorous prospective data collection framework, we implemented a comprehensive quality assurance protocol to ensure data integrity throughout the 24-month follow-up period. Data were collected prospectively using standardized electronic case report forms designed specifically for this study and implemented uniformly across all five participating centers. Three complementary data sources ensured completeness and accuracy: real-time clinical data entry by treating gastroenterologists at each study visit, centralized laboratory records for all biomarker measurements, including IL-8, CEA, and CA19-9, and independent histopathology reports from two blinded pathologists with reconciliation records. Cross-referencing these sources enabled complete ascertainment of all protocol-required data elements for enrolled participants. Rigorous quality control measures included double data entry for 10% of randomly selected records with greater than 98% concordance required, systematic source data verification against original endoscopy and pathology reports for all progression cases, monthly monitoring reports from each site reviewed by the coordinating center, and documentation of missingness rates for all key variables with prespecified handling in the statistical analysis plan.

### Missing data management

Missing data patterns were examined using Little’s Missing Completely at Random test. For IL-8 measurements, if a single time point was missing, imputation using last observation carried forward was considered only if 20% or fewer of the time points were missing. Patients with more than 20% missing IL-8 measurements were excluded from trajectory analyses but were included in baseline cross-sectional analyses where applicable. For covariates with less than 10% missingness, multiple imputation by chained equations was performed with 20 imputed datasets, and results were pooled according to Rubin’s rules.

### Statistical analysis

Statistical analyses were performed using R version 4.2.2 (R Foundation for Statistical Computing, Vienna, Austria) with additional packages including lme4 for mixed-effects models, traj for group-based trajectory modeling, pROC for receiver operating characteristic curve analysis, and survival for time-to-event analyses. Two-sided p-values less than 0.05 were considered statistically significant. No adjustments for multiple comparisons were made for secondary outcomes, which were considered exploratory.

Continuous variables were summarized as mean ± standard deviation for normally distributed data or median with interquartile range for non-normally distributed data. Normality was assessed using the Shapiro-Wilk test and visual inspection of Q-Q plots. Categorical variables were presented as frequencies and percentages. Baseline characteristics were compared across groups using analysis of variance with post-hoc Tukey correction for pairwise comparisons for normally distributed continuous variables, Kruskal-Wallis test for non-normally distributed variables, and chi-square test or Fisher’s exact test for categorical variables as appropriate.

Differences in IL-8 levels across the five groups were assessed using one-way analysis of variance with linear trend analysis to test for progressive increase across the histological spectrum. Pairwise comparisons between adjacent groups were performed using independent t-tests with Bonferroni correction for multiple comparisons.

For longitudinal analyses, linear mixed-effects models were employed to model IL-8 trajectories over the 24-month follow-up period. The model included fixed effects for time as a continuous variable, group, and their interaction, with random intercepts and random slopes for time at the patient level to account for within-subject correlation. An unstructured covariance matrix was specified. The primary parameter of interest was the time-by-progression interaction, testing whether IL-8 trajectories differed between patients who progressed to cancer and those who did not.

Group-based trajectory modeling was performed using the traj package to identify distinct latent classes of IL-8 trajectories over 24 months. This exploratory analysis aimed to identify potential patterns of IL-8 evolution that could inform future risk stratification models. Models with two to five trajectory groups and linear, quadratic, and cubic polynomials were compared. A censored normal distribution was specified for the trajectory models, appropriate for the continuous and approximately normally distributed IL-8 measurements. The optimal number of groups and polynomial order was selected based on Bayesian Information Criterion, average posterior probability of group membership (> 0.7 for all groups), and minimum class size of at least 5% of the sample (*n* ≥ 5) to ensure clinical interpretability. Crucially, trajectory classes were derived without any knowledge of patient outcome status (progression vs. non-progression); outcome data were merged only after the final trajectory solution was determined. Model estimation was performed blinded to outcome status to prevent circularity. To assess model stability and quantify uncertainty in trajectory assignments, we calculated entropy (average posterior probability of assignment) and performed bootstrap resampling with 500 replications to generate 95% confidence intervals around trajectory probabilities. As a sensitivity analysis, we repeated trajectory modeling after randomly splitting the cohort into derivation (70%) and validation (30%) samples to assess the reproducibility of the three-class solution.

Receiver operating characteristic curve analysis was performed to evaluate the ability of baseline IL-8 to predict progression to gastric cancer. The area under the receiver operating characteristic curve with 95% confidence intervals was calculated using the DeLong method. The optimal cut-off value was determined by maximizing the Youden index. Sensitivity, specificity, positive predictive value, negative predictive value, positive likelihood ratio, and negative likelihood ratio were calculated at this cut-off. The stability of the optimal cut-off value was assessed using bootstrap resampling with 1000 iterations, recalculating the Youden index-derived cut-off in each bootstrap sample and generating 95% confidence intervals around the cut-off estimate. Eradication status was not included as a time-varying covariate in the primary Cox model, as sensitivity analysis demonstrated no significant differences in IL-8 trajectories between successfully eradicated and persistently infected patients (*p* = 0.34), indicating that eradication did not confound the association between IL-8 and progression.

Time-to-event analysis. To evaluate the predictive value of baseline IL-8 while avoiding reverse causality, Cox proportional hazards regression was performed to estimate hazard ratios (HR) for progression to gastric cancer. Patients without progression were censored at the time of last follow-up endoscopy (24 months) or at the time of death from unrelated causes. Kaplan–Meier survival curves were constructed stratified by baseline IL-8 level using the optimal cut-off of 52.3 pg/mL derived from ROC analysis, and differences between curves were assessed using the log-rank test. Given the limited number of progression events (*n* = 9), the Cox model was restricted to baseline IL-8 as the primary predictor, with adjustment for histopathological group (intestinal metaplasia vs. atrophic gastritis) as a prespecified covariate.

Time-dependent receiver operating characteristic analysis was conducted using the Kaplan-Meier estimator to assess the predictive accuracy of IL-8 measured at baseline, 6, 12, and 18 months for progression occurring within the subsequent follow-up period. Cumulative and dynamic areas under the curve were calculated at each time point.

Comparative performance between IL-8, carcinoembryonic antigen, and carbohydrate antigen 19 − 9 was assessed by comparing areas under the curve using the DeLong test for correlated receiver operating characteristic curves. Net reclassification improvement and integrated discrimination improvement were calculated to quantify the incremental value of adding IL-8 to a baseline model containing conventional risk factors, including age, sex, and histopathological group.

Univariate logistic regression analysis was performed to identify potential predictors of progression. Given the limited number of progression events (*n* = 9), the number of covariates included in multivariable models was restricted to minimize overfitting, consistent with recommended events-per-variable considerations. Therefore, multivariable analysis was restricted to a maximum of two clinically justified predictors: baseline IL-8 and histopathological group (intestinal metaplasia vs. atrophic gastritis), selected a priori based on biological plausibility and univariate findings. This restriction was applied deliberately to avoid model overfitting, given the limited number of progression events (*n* = 9), consistent with recommended events-per-variable considerations in the statistical literature [27, 28]. Additional covariates (including age, sex, hemoglobin, CEA, and CA19-9) were evaluated in univariate analyses (Table 7) but were not included in the final multivariable model to preserve model stability and interpretability, as their inclusion would risk unstable estimates and artificially wide confidence intervals. To further address concerns related to sparse data, we employed Firth’s penalized likelihood logistic regression, which reduces bias in maximum likelihood estimates for small sample sizes [Huang, 2021]. Results were reported as penalized odds ratios with profile likelihood confidence intervals. Internal validation was performed using bootstrap resampling with 1000 iterations to estimate the optimism-corrected C-statistic and calibration slope [27]. For stage-stratified comparisons of IL-8 levels between *H. pylori*-positive and *H. pylori*-negative gastric cancer patients presented in Table 3, we employed independent t-tests with the understanding that small subgroup sizes (n as low as 4 per group) limit statistical power; these analyses are considered exploratory.

For the exploratory predictive models comparing IL-8 with clinical/histopathological risk factors, all logistic regression models were fitted using Firth’s penalized likelihood method to reduce small-sample bias and mitigate separation associated with the limited number of progression events (*n* = 9). Given the small number of progression events, the reported AUC estimates may be subject to substantial optimism and should not be interpreted as evidence of a validated prediction model. Formal calibration assessment was considered unreliable given the small number of progression events; therefore, we have not presented calibration estimates that could give a misleading impression of model stability. These model comparisons are exploratory, and the results are presented as evidence of incremental discrimination rather than definitive validation.

Pre-specified subgroup analyses were performed according to histopathological group, age, sex, center, and smoking status. Sensitivity analyses were conducted to assess the robustness of findings, including complete case analysis excluding patients with any missing IL-8 measurements, multiple imputation for missing IL-8 values using chained equations with 20 imputed datasets, exclusion of patients with protocol violations or inadequate biopsy sampling, and analysis using alternative IL-8 cut-off values within 10% of the optimal cut-off.

To assess potential inter-center and inter-operator variability, we performed sensitivity analyses including center as a random effect in mixed models and formally tested for center-by-treatment interactions. No significant center effects were detected (*p* > 0.20 for all comparisons), supporting the robustness and generalizability of our findings across different clinical settings and operators.

Internal validation was performed for all key predictive analyses. For the baseline IL-8 ROC analysis, we calculated optimism-corrected AUC using 1000 bootstrap samples with the 0.632 estimator [28]. For the multivariable Firth logistic regression model, bootstrap resampling with 1000 iterations was used to estimate optimism-corrected C-statistic and calibration slope, with calibration assessed by the Hosmer-Lemeshow test on bootstrap samples. For trajectory modeling, as described above, bootstrap resampling with 500 replications generated confidence intervals around trajectory probabilities, and a 70/30 split-sample sensitivity analysis was performed to assess reproducibility. These internal validation procedures provide reassurance regarding the stability and generalizability of our findings, though external validation in independent cohorts remains necessary. All statistical analyses were performed by a qualified biostatistician blinded to group assignments, and results were independently verified by a second statistician to ensure accuracy and reproducibility.

Sensitivity to unmeasured confounding was assessed using the E-value approach [29]. The E-value was calculated for the primary adjusted hazard ratio and for the confidence limit closest to the null to quantify the minimum strength of association that an unmeasured confounder would require with both elevated baseline IL-8 and progression to gastric cancer to explain the observed association, conditional on the measured covariates.

## Results

### Study population and baseline characteristics

A total of 200 participants were enrolled across the five participating centers, comprising 50 patients with atrophic gastritis (Group I), 50 patients with intestinal metaplasia (Group II), 25 patients with *H. pylori*-positive gastric cancer (Group III), 25 patients with *H. pylori*-negative gastric cancer (Group IV), and 50 healthy controls (Group V). All 200 participants completed baseline evaluations, including endoscopy with biopsy, histopathological classification, and *H. pylori* testing using stool antigen and urea breath test, and were included in the baseline cross-sectional analyses (the 5-group comparison). The 100 patients with precancerous lesions (Groups I and II) were subsequently followed longitudinally for 24 months.

Among the 125 patients classified as *H. pylori*-positive (Groups I, II, and III), 117 (93.6%) had concordant positive results on both tests at initial screening. The remaining 8 patients (6.4%) had discordant results (stool antigen positive/UBT negative or vice versa) and underwent histopathological examination of gastric biopsies with modified Giemsa staining. All 8 were confirmed to have active *H. pylori* infection with associated gastritis and were included in the positive cohort. No patients required exclusion due to unresolved diagnostic discordance.

For the longitudinal cohort, the 100 patients with precancerous lesions (Groups I and II) eligible for 24-month follow-up, 94 patients (94%) completed the full study protocol, while 6 patients (6%) were lost to follow-up (4 from the atrophic gastritis group and 2 from the intestinal metaplasia group). Reasons for loss to follow-up included relocation to another governorate (*n* = 3), withdrawal of consent (*n* = 2), and death from unrelated causes (*n* = 1). Importantly, there were no significant differences in baseline characteristics between patients who completed follow-up and those who were lost to follow-up (*p* > 0.20 for all comparisons), minimizing the risk of attrition bias.

Table 1 presents the baseline demographic and clinical characteristics of the study population stratified by group. The mean age of the entire cohort was 54.3 ± 12.7 years, with significant differences across groups (*p* < 0.001). Patients in the gastric cancer groups were significantly older (mean 62.4 ± 8.9 years for *H. pylori*-positive and 61.8 ± 9.3 years for *H. pylori*-negative) compared to healthy controls (44.2 ± 10.1 years), while patients with precancerous lesions had intermediate ages (atrophic gastritis: 52.8 ± 9.7 years; intestinal metaplasia: 57.6 ± 8.4 years). Male predominance was observed across all disease groups, with the highest proportion in gastric cancer patients (68% in *H. pylori*-positive and 64% in *H. pylori*-negative groups), compared to 48% in healthy controls (*p* = 0.04 for sex distribution across groups).

Conventional laboratory parameters demonstrated expected patterns across the disease spectrum, reflecting the systemic impact of gastric carcinogenesis. Hemoglobin levels decreased progressively from healthy controls (13.6 ± 1.1 g/dL) to atrophic gastritis (12.5 ± 0.9 g/dL), intestinal metaplasia (12.1 ± 1.2 g/dL), and gastric cancer groups (9.8 ± 1.3 g/dL for *H. pylori*-positive and 10.2 ± 1.4 g/dL for *H. pylori*-negative), with the differences being highly significant (*p* < 0.001). Similarly, serum albumin showed a declining trend across groups, with the lowest values observed in gastric cancer patients (3.6 ± 0.4 g/dL in *H. pylori*-positive and 3.7 ± 0.5 g/dL in *H. pylori*-negative groups) compared to healthy controls (4.4 ± 0.3 g/dL, *p* < 0.001). White blood cell counts were elevated in gastric cancer patients compared to other groups (mean 8.9 ± 2.1 × 10³/µL in cancer groups vs. 7.2 ± 1.8 × 10³/µL in non-cancer groups, *p* < 0.001), reflecting the systemic inflammatory response associated with malignancy.

Other baseline characteristics, including BMI, smoking status, diabetes prevalence, and platelet counts, were balanced across groups or showed only minor differences that did not reach statistical significance (all *p* > 0.05), except creatinine, which was slightly higher in gastric cancer patients (*p* < 0.001), likely reflecting age-related changes and systemic effects of malignancy.

Conventional tumor markers showed limited sensitivity for early detection. Carcinoembryonic antigen and carbohydrate antigen 19 − 9 were within normal ranges in the majority of healthy controls and patients with precancerous lesions, but were significantly elevated in gastric cancer patients. Median carcinoembryonic antigen levels were 1.8 ng/mL (interquartile range 1.2–2.6) in healthy controls, compared to 32.5 ng/mL (interquartile range 18.7–48.3) in *H. pylori*-positive gastric cancer and 28.9 ng/mL (interquartile range 15.2–44.6) in *H. pylori*-negative gastric cancer (*p* < 0.001). Similarly, median carbohydrate antigen 19 − 9 levels were 13.5 U/mL (interquartile range 9.2–18.1) in healthy controls, compared to 128.6 U/mL (interquartile range 86.2-168.4) in *H. pylori*-positive gastric cancer and 119.4 U/mL (interquartile range 78.3-158.7) in *H. pylori*-negative gastric cancer (*p* < 0.001). However, considerable overlap existed between groups, with 32% of gastric cancer patients having carcinoembryonic antigen levels below 5 ng/mL and 28% having carbohydrate antigen 19 − 9 levels below 37 U/mL, highlighting the limited sensitivity of conventional markers for early detection and underscoring the need for more accurate biomarkers such as IL-8.

**Fig. 1:**
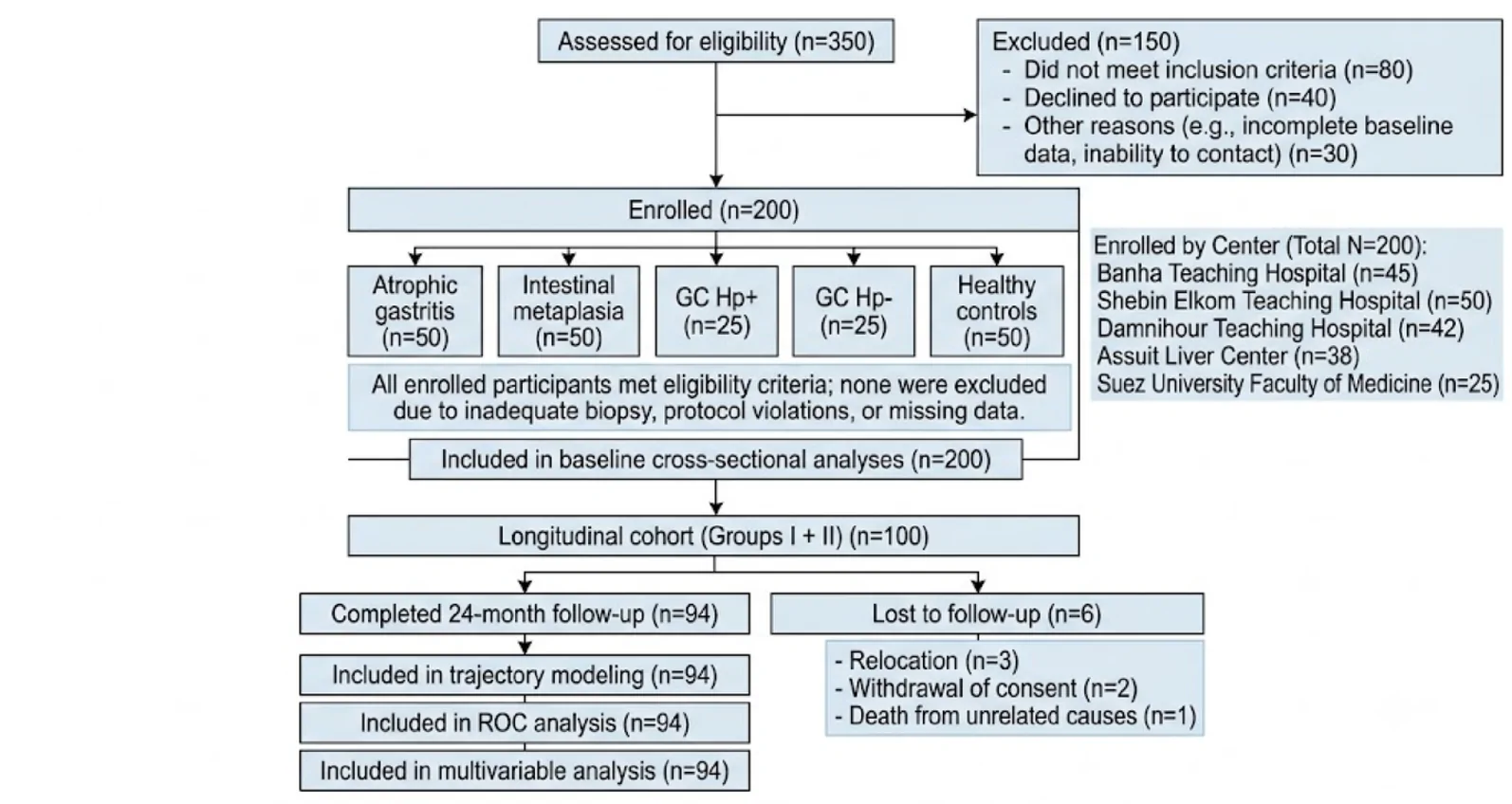
Participant flow diagram. A total of 350 individuals were assessed for eligibility, of whom 150 were excluded (80 did not meet inclusion criteria, 40 declined participation, and 30 were excluded for other reasons, including incomplete baseline data or inability to contact). The remaining 200 participants were enrolled across five study centers and included in baseline cross-sectional analyses. Among the 100 patients with precancerous lesions enrolled in the longitudinal cohort (Groups I and II), 94 (94%) completed the 24-month follow-up and were included in all longitudinal analyses (trajectory modeling, ROC analysis, and multivariable analysis). No patients were excluded from analyses due to inadequate biopsy sampling, protocol violations, or missing IL-8 measurements. GC: gastric cancer; Hp+: *H. pylori*-positive; Hp-: *H. pylori*-negative

Figure 1 presents the participant flow diagram. A total of 350 individuals were assessed for eligibility, of whom 150 were excluded (80 did not meet inclusion criteria, 40 declined participation, and 30 were excluded for other reasons, including incomplete baseline data or inability to contact). The remaining 200 participants were enrolled across the five study centers: Banha Teaching Hospital (*n* = 45), Shebin Elkom Teaching Hospital (*n* = 50), Damnhour Teaching Hospital (*n* = 42), Assuit Liver Center (*n* = 38), and Suez University Faculty of Medicine (*n* = 25). All 200 participants were included in the baseline cross-sectional analyses. Of the 100 patients with precancerous lesions enrolled in the longitudinal cohort, 94 (94%) completed the 24-month follow-up and were included in all longitudinal analyses (trajectory modeling, ROC analysis, and multivariable analysis). No patients were excluded from analyses due to inadequate biopsy sampling, protocol violations, or missing IL-8 measurements.

**Table 1:** Baseline Demographic and Clinical Characteristics of the Study Population

Characteristic	Healthy Controls (*n* = 50)	Atrophic Gastritis (*n* = 50)	Intestinal Metaplasia (*n* = 50)	GC Hp+ (*n* = 25)	GC Hp- (*n* = 25)	*p*-value
Age, years	44.2 ± 10.1	52.8 ± 9.7	57.6 ± 8.4	62.4 ± 8.9	61.8 ± 9.3	< 0.001
Sex, male (%)	24 (48%)	28 (56%)	31 (62%)	17 (68%)	16 (64%)	0.04
BMI, kg/m²	26.8 ± 3.4	25.9 ± 3.7	26.2 ± 4.1	24.1 ± 3.8	24.5 ± 4.0	0.02
Smoking, current (%)	12 (24%)	16 (32%)	19 (38%)	11 (44%)	10 (40%)	0.12
Diabetes mellitus (%)	6 (12%)	9 (18%)	11 (22%)	7 (28%)	6 (24%)	0.28
Hemoglobin, g/dL	13.6 ± 1.1	12.5 ± 0.9	12.1 ± 1.2	9.8 ± 1.3	10.2 ± 1.4	< 0.001
WBC, ×10³/µL	6.8 ± 1.5	7.4 ± 1.9	7.9 ± 2.1	8.8 ± 2.3	8.6 ± 2.0	< 0.001
Platelets, ×10³/µL	268 ± 52	274 ± 61	258 ± 58	242 ± 67	251 ± 63	0.08
Albumin, g/dL	4.4 ± 0.3	4.2 ± 0.4	4.1 ± 0.4	3.6 ± 0.4	3.7 ± 0.5	< 0.001
Creatinine, mg/dL	0.82 ± 0.14	0.96 ± 0.18	0.94 ± 0.16	1.08 ± 0.22	1.04 ± 0.19	< 0.001
CEA, ng/mL, median (IQR)	1.8 (1.2–2.6)	2.9 (2.1–3.8)	3.8 (2.8–5.1)	32.5 (18.7–48.3)	28.9 (15.2–44.6)	< 0.001
CA19-9, U/mL, median (IQR)	13.5 (9.2–18.1)	26.4 (18.3–34.2)	31.8 (22.7–42.6)	128.6 (86.2-168.4)	119.4 (78.3-158.7)	< 0.001

Data are presented as mean ± standard deviation unless otherwise indicated. P-values were calculated using ANOVA for continuous variables with normal distribution, the Kruskal-Wallis test for non-normally distributed variables, and the chi-square test for categorical variables. GC Hp+: gastric cancer, *H. pylori*-positive; GC Hp-: gastric cancer, *H. pylori*-negative; BMI: body mass index; WBC: white blood cells; CEA: carcinoembryonic antigen; CA19-9: carbohydrate antigen 19 − 9; IQR: interquartile range. Groups I and II were *H. pylori*-positive at baseline and received eradication therapy after enrollment. Group labels reflect baseline status

### Histopathological confirmation of study groups

Histopathological evaluation of endoscopic biopsy specimens confirmed the accuracy of group classification for all 200 participants according to the Updated Sydney System criteria. Representative photomicrographs illustrating the sequential histological spectrum from normal gastric mucosa to gastric adenocarcinoma are presented in Fig. 1 (A–E).

**Fig. 2:**
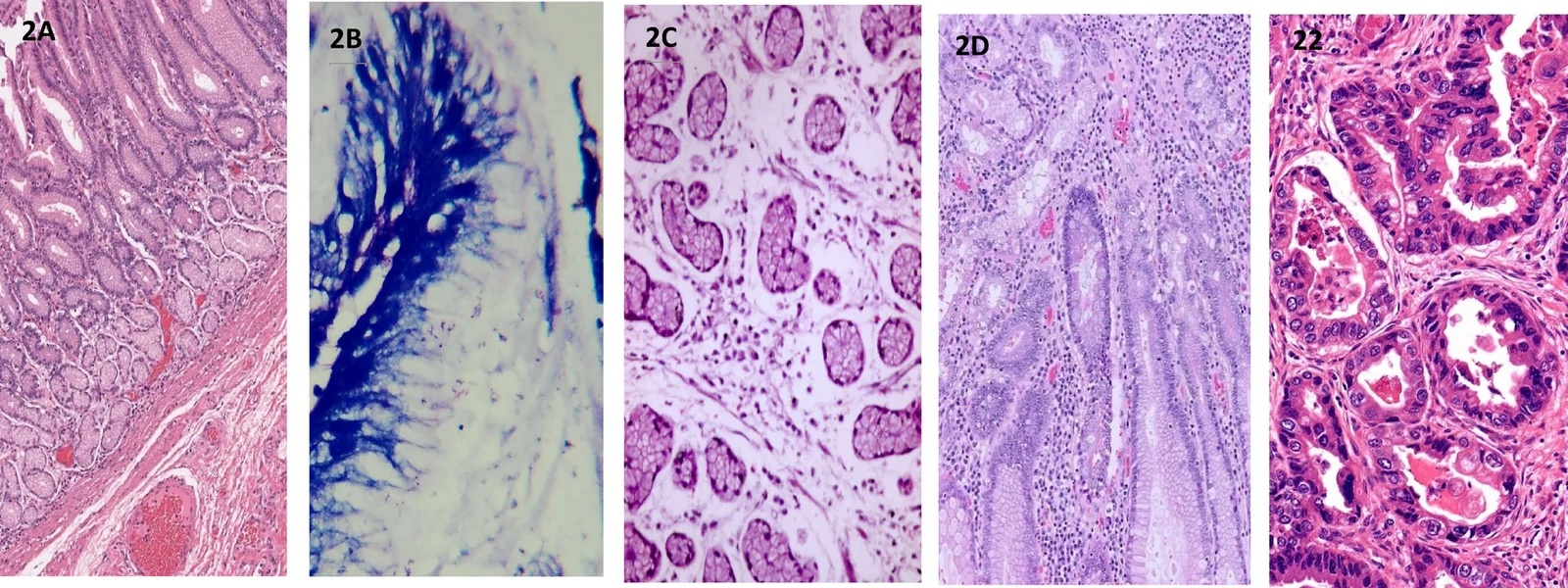
Histopathological spectrum of gastric carcinogenesis. Representative photomicrographs from study participants. (**A**) Normal gastric mucosa (hematoxylin and eosin [H&E], ×200) showing intact foveolar epithelium with preserved glandular architecture and absence of inflammatory infiltrates, atrophy, intestinal metaplasia, dysplasia, or Helicobacter pylori. (**B**) Helicobacter pylori infection demonstrated by modified Giemsa stain (×400), showing curved bacilli adherent to the surface epithelium and within the mucus layer. (**C**) Chronic atrophic gastritis (H&E, ×400) characterized by glandular loss, reduced gland density, and expansion of the lamina propria by chronic inflammatory infiltrates composed predominantly of lymphocytes and plasma cells. (**D**) Intestinal metaplasia (H&E, ×200) showing replacement of native gastric epithelium by intestinal-type epithelium with goblet cells and absorptive columnar cells. (**E**) Gastric adenocarcinoma (H&E, ×200) demonstrating infiltrative malignant glands with nuclear pleomorphism and hyperchromasia within a desmoplastic stromal background

All healthy controls (Group V) demonstrated normal gastric mucosa with intact glandular architecture and no evidence of Helicobacter pylori infection **(**Fig. 2A**)**. The foveolar epithelium exhibited preserved cellular polarity, and the lamina propria was unremarkable without inflammatory infiltration, atrophy, metaplasia, or dysplasia.

All patients in Groups I–III had histologically confirmed active *H. pylori* infection. Organisms were visualized on modified Giemsa staining as curved bacilli adherent to the surface epithelium and within the mucus layer **(**Fig. 2B**)**. Among *H. pylori*–positive patients, bacterial density scores did not differ significantly between the atrophic gastritis, intestinal metaplasia, and gastric cancer groups (median density grade 2, interquartile range 1–3; *p* = 0.34). Patients with chronic atrophic gastritis (Group I) demonstrated glandular loss with reduced gland density and expansion of the lamina propria by chronic inflammatory infiltrates composed predominantly of lymphocytes and plasma cells (median atrophy grade 2; Fig. 2C). Patients with intestinal metaplasia (Group II) showed replacement of native gastric epithelium by intestinal-type epithelium characterized by goblet cells containing mucin vacuoles and absorptive columnar cells **(**Fig. 2D**)**. Among these patients, 42% exhibited (type III) intestinal metaplasia, the subtype associated with the highest malignant potential. Patients with metaplasia had significantly higher baseline IL-8 levels compared to those with complete (type I) metaplasia (71.4 ± 12.8 vs. 58.2 ± 11.6 pg/mL; mean difference 13.2 pg/mL; 95% CI: 7.1–19.3; *p* < 0.001). All gastric cancer patients (Groups III and IV) had histologically confirmed adenocarcinoma. According to Lauren’s classification, 76% were intestinal-type, and 24% were diffuse-type. Histologically, tumors demonstrated infiltrative malignant glands composed of atypical epithelial cells with nuclear pleomorphism and hyperchromasia within a desmoplastic stromal background **(**Fig. 2E**)**. A significant association was observed between histological subtype and background mucosa: 82% of intestinal-type cancers arose in mucosa with extensive intestinal metaplasia, compared to 25% of diffuse-type cancers (*p* < 0.001).

### Cross-sectional analysis of serum IL-8 across the gastric carcinogenesis spectrum

**Fig. 3:**
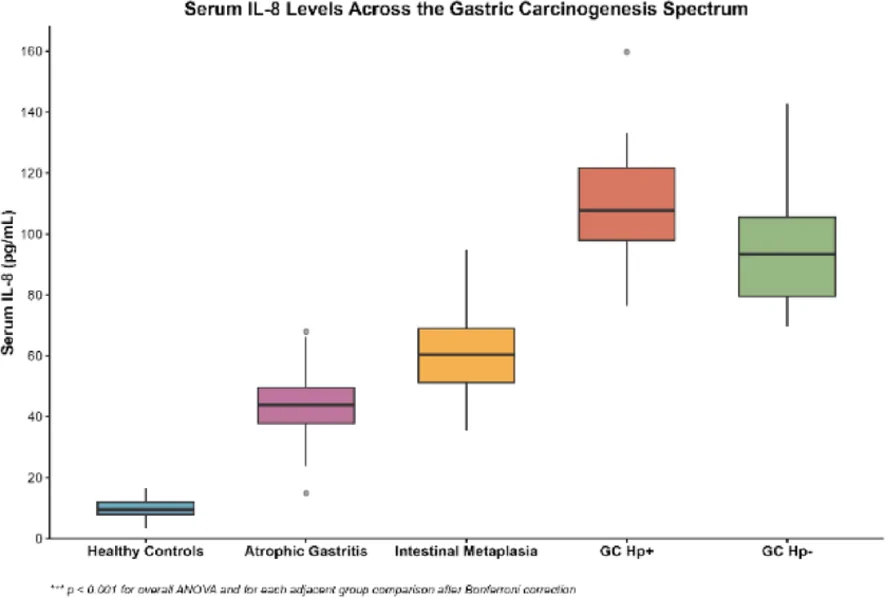
Serum IL-8 Levels Across the Gastric Carcinogenesis Spectrum. Box plots depict the median (horizontal line), interquartile range (box), and whiskers extending to 1.5× the interquartile range of serum IL-8 concentrations in healthy controls (n=50), atrophic gastritis (n=50), intestinal metaplasia (n=50), H. pylori-positive gastric cancer (n=25), and H. pylori-negative gastric cancer (n=25). ***P < 0.001 for overall one-way ANOVA and for pairwise adjacent group comparisons following Bonferroni correction

To establish the relationship between IL-8 and gastric carcinogenesis across the full disease spectrum, including both *H. pylori*-positive and *H. pylori*-negative cancers, we first performed cross-sectional comparisons among all five study groups. Serum IL-8 levels demonstrated a striking progressive increase across the histological spectrum from normal gastric mucosa through atrophic gastritis and intestinal metaplasia to gastric cancer, as illustrated in Fig. 3 and detailed in Table 2.

Mean baseline IL-8 concentrations were lowest in healthy controls (9.7 ± 3.2 pg/mL), increased more than four-fold in patients with atrophic gastritis (42.1 ± 11.8 pg/mL), rose further in intestinal metaplasia (64.8 ± 14.3 pg/mL), and reached levels approximately 10-fold higher than controls in gastric cancer patients (107.4 ± 24.6 pg/mL in *H. pylori*-positive and 98.6 ± 22.1 pg/mL in *H. pylori*-negative groups). One-way analysis of variance revealed highly significant differences across groups (F = 184.3, *p* < 0.001), and the test for linear trend confirmed a monotonic increase across the disease spectrum (*p* for trend < 0.001).

Pairwise comparisons confirmed that each step along the carcinogenesis cascade was associated with a significant increase in IL-8 levels (all *p* < 0.001). As shown in Table 2, the transition from healthy mucosa to atrophic gastritis represented the largest relative increase (more than 300% elevation), while the transition from intestinal metaplasia to gastric cancer showed the largest absolute increase (mean difference 42.6 pg/mL for *H. pylori*-positive cancer). Notably, IL-8 levels did not differ significantly between *H. pylori*-positive and *H. pylori*-negative gastric cancer patients (mean difference 8.8 pg/mL, 95% CI: -4.2 to 21.8, *p* = 0.18).

Effect size calculations using Cohen’s d revealed large differences between groups. The comparison between healthy controls and atrophic gastritis yielded a Cohen’s d of 3.42 (95% CI: 2.84-4.00). The difference between intestinal metaplasia and gastric cancer yielded a Cohen’s d of 2.18 (95% CI: 1.72–2.64). For comparison, Cohen’s d for the difference between intestinal metaplasia and gastric cancer was 0.94 for carcinoembryonic antigen and 0.86 for carbohydrate antigen 19 − 9.

**Table 2:** Serum IL-8 Levels Across Disease Groups with Pairwise Comparisons

Group	*n*	IL-8 (pg/mL) Mean ± SD	Comparison	Mean Difference (95% CI)	*p*-value*	Cohen’s d (95% CI)
Healthy controls	50	9.7 ± 3.2	Reference	—	—	—
Atrophic gastritis	50	42.1 ± 11.8	vs. Controls	32.4 (27.8–37.0)	< 0.001	3.42 (2.84-4.00)
Intestinal metaplasia	50	64.8 ± 14.3	vs. AG	22.7 (17.9–27.5)	< 0.001	1.72 (1.35–2.09)
GC Hp+	25	107.4 ± 24.6	vs. IM	42.6 (35.8–49.4)	< 0.001	2.18 (1.72–2.64)
GC Hp-	25	98.6 ± 22.1	vs. IM	33.8 (27.1–40.5)	< 0.001	1.84 (1.40–2.28)
GC Hp + vs. GC Hp-	—	—	—	8.8 (-4.2 to 21.8)	0.18	0.38 (-0.18 to 0.94)

*P-values calculated using independent t-tests with Bonferroni correction for multiple comparisons (adjusted α = 0.0125). Overall ANOVA F = 184.3, *p* < 0.001. AG: atrophic gastritis; IM: intestinal metaplasia; GC Hp+: gastric cancer, *H. pylori*-positive; GC Hp-: gastric cancer, *H. pylori*-negative; SD: standard deviation; CI: confidence interval

### IL-8 levels in gastric cancer: independence from *H. pylori* infection status

IL-8 levels were compared between *H. pylori*-positive and *H. pylori*-negative gastric cancer patients stratified by tumor stage **(**Table 3**)**. At each stage, no significant differences in IL-8 levels were observed between infection-positive and infection-negative patients, with mean differences ranging from 3.2 to 4.4 pg/mL, and all p-values > 0.50. These stage-stratified analyses were exploratory due to small sample sizes within individual stage groups (particularly stage IV, *n* = 8 total).

**Fig. 4:**
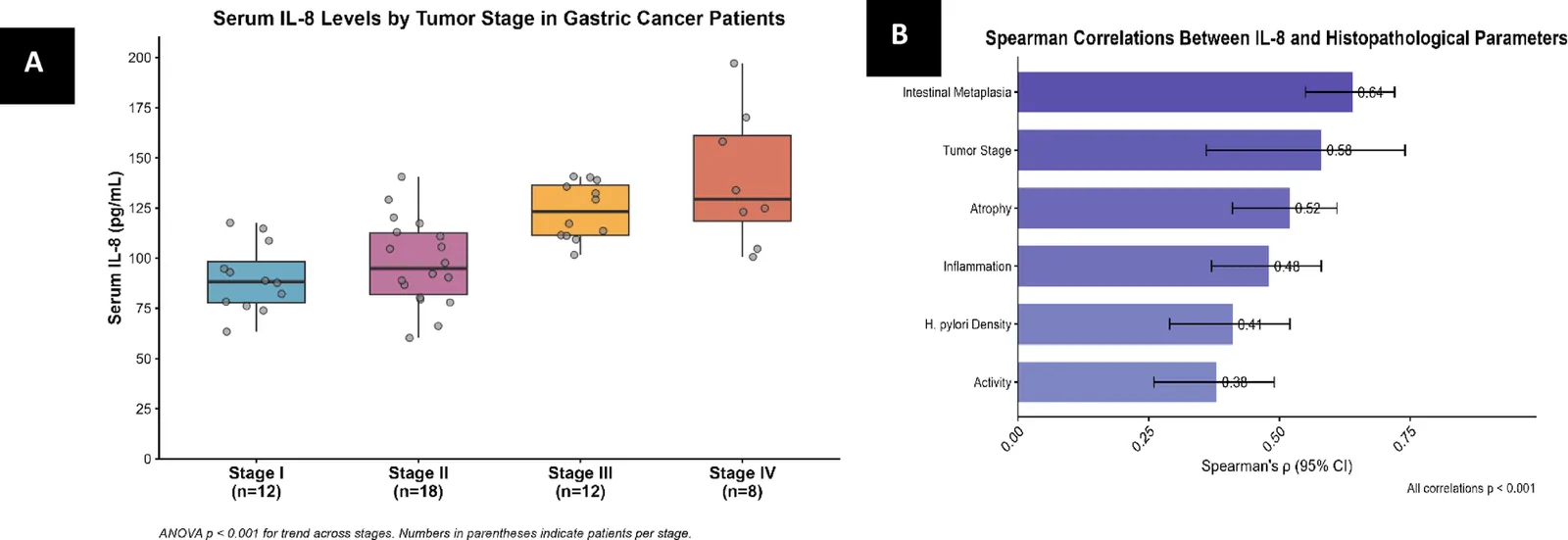
Correlation of Serum IL-8 with Histopathological Parameters and Tumor Stage. (**A**) Serum IL-8 levels in gastric cancer patients stratified by AJCC 8th edition stage. Box plots depict median, interquartile range, and whiskers extending to 1.5× IQR with individual data points overlaid. IL-8 increases progressively with stage (ANOVA *p* < 0.001). Numbers in parentheses indicate patients per stage. (**B**) Spearman correlation coefficients (ρ) with 95% confidence intervals for associations between serum IL-8 and histopathological features assessed by the Updated Sydney System. All correlations were significant (*p* < 0.001). Intestinal metaplasia grade showed the strongest correlation (ρ = 0.64), followed by tumor stage (ρ = 0.58), atrophy (ρ = 0.52), inflammation (ρ = 0.48), *H. pylori* density (ρ = 0.41), and activity (ρ = 0.38)

As illustrated in Fig. 4A, a progressive increase in IL-8 levels was observed with advancing tumor stage within each infection-status group. Mean IL-8 levels increased from stage I to stage IV in both *H. pylori*-positive and *H. pylori*-negative patients (*p* for trend < 0.001 for both groups).

**Table 3:** Serum IL-8 Levels in Gastric Cancer Patients Stratified by Stage and *H. pylori* Status

Tumor Stage	Hp + GC (*n* = 25)	Hp- GC (*n* = 25)	Mean Difference (95% CI)	*p*-value*
Stage I (*n* = 12)	*n* = 7, 88.2 ± 17.6	*n* = 5, 84.6 ± 18.9	3.6 (-12.4 to 19.6)	0.62
Stage II (*n* = 18)	*n* = 10, 104.1 ± 20.3	*n* = 8, 100.5 ± 22.1	3.6 (-15.8 to 23.0)	0.58
Stage III (*n* = 12)	*n* = 5, 120.3 ± 23.8	*n* = 7, 117.1 ± 25.4	3.2 (-24.1 to 30.5)	0.71
Stage IV (*n* = 8)	*n* = 3, 138.4 ± 27.2	*n* = 5, 134.0 ± 29.3	4.4 (-36.2 to 45.0)	0.69
All stages	*n* = 25,** 107.4 ± 24.6**	**98.6 ± 22.1**	**8.8 (-4.2 to 21.8)**	**0.18**

Data presented as mean ± SD pg/mL. *P-values from independent t-tests comparing Hp + and Hp- groups within each stage. Hp+: *H. pylori*-positive; Hp-: *H. pylori*-negative; GC: gastric cancer; CI: confidence interval. AJCC 8th edition staging is used for all patients based on endoscopic ultrasound and cross-sectional imaging when available. These stage-stratified analyses were exploratory and were not adjusted for multiple comparisons, consistent with the hypothesis-generating nature of this investigation.Stage distribution: Stage I (Hp + *n* = 7, Hp- *n* = 5); Stage II (Hp + *n* = 10, Hp- *n* = 8); Stage III (Hp + *n* = 5, Hp- *n* = 7); Stage IV (Hp + *n* = 3, Hp- *n* = 5)

### Correlation of IL-8 with histopathological parameters

To elucidate the pathological determinants of circulating IL-8 levels, we examined correlations between serum IL-8 and detailed histopathological features assessed by the Updated Sydney System. As shown in Table 4, serum IL-8 correlated significantly with all histopathological parameters, most strongly with intestinal metaplasia grade. Among patients with intestinal metaplasia, those with incomplete (type III) subtype had significantly higher IL-8 levels than those with complete (type I) subtype (mean difference 13.2 pg/mL, 95% CI: 7.1–19.3, *p* < 0.001), indicating that IL-8 reflects the biological aggressiveness of metaplasia. In gastric cancer patients, IL-8 levels correlated significantly with tumor stage **(**Fig. 4**)**, with progressive increases from stage I through stage IV (ANOVA *p* < 0.001). No significant difference in IL-8 levels was observed between intestinal-type and diffuse-type cancers (*p* = 0.38).

**Table 4:** Correlation Between Serum IL-8 and Histopathological Parameters

Parameter	Spearman’s ρ	95% CI	*p*-value
Intestinal metaplasia grade	0.64	0.55–0.72	< 0.001
Atrophy grade	0.52	0.41–0.61	< 0.001
Inflammation grade	0.48	0.37–0.58	< 0.001
*H. pylori* density	0.41	0.29–0.52	< 0.001
Activity grade (neutrophilic infiltration)	0.38	0.26–0.49	< 0.001
Tumor stage (cancer patients only)	0.58	0.36–0.74	< 0.001

CI: confidence interval. Correlations were calculated using Spearman’s rank correlation coefficient across all 200 participants unless otherwise specified. Given the exploratory nature of these correlation analyses, no adjustment for multiple testing was applied, in accordance with our prespecified analytical plan

### Association of IL-8 with baseline patient characteristics and incremental predictive value

We examined the association between baseline IL-8 levels and established gastric cancer risk factors. IL-8 levels were positively correlated with age (ρ = 0.31, *p* < 0.001), higher in males than females (52.4 ± 15.2 vs. 44.7 ± 14.1 pg/mL, *p* = 0.02), and higher in current smokers than non-smokers (56.3 ± 16.4 vs. 47.8 ± 13.6 pg/mL, *p* = 0.04). No significant associations were observed with BMI (ρ = -0.08, *p* = 0.31) or diabetes (51.2 ± 15.8 vs. 49.6 ± 14.4 pg/mL, *p* = 0.42). IL-8 levels were significantly higher in *H. pylori*-positive patients than *H. pylori*-negative patients (54.6 ± 15.1 vs. 39.8 ± 12.3 pg/mL, *p* < 0.001).

To evaluate the incremental predictive value of IL-8 beyond established risk factors, we compared a model based on clinical and histopathological risk factors alone (age, sex, smoking status, and intestinal metaplasia/atrophy) with a model incorporating IL-8. *H. pylori* status was not included in the longitudinal prediction model because all patients with precancerous lesions were *H. pylori*-positive at baseline; however, the cross-sectional analysis **(**Table 3**)** showed that IL-8 levels remained elevated among patients with gastric cancer irrespective of H. pylori infection status. The addition of IL-8 significantly improved the AUC from 0.68 (95% CI: 0.58–0.78) to 0.85 (95% CI: 0.77–0.93) (*p* < 0.001). These model comparisons are exploratory; all models were fitted using Firth’s penalized likelihood method to reduce small-sample bias and mitigate separation associated with the limited number of progression events (*n* = 9). The results are presented as evidence of incremental discrimination rather than definitive validation.

### OLGA/OLGIM staging and association with IL-8

Using the systematic biopsies obtained from the antrum, incisura angularis, and corpus according to the Updated Sydney System, we calculated OLGA and OLGIM stages for the 94 patients with precancerous lesions. Serum IL-8 levels increased progressively with both OLGA and OLGIM stage: from 32.1 ± 8.4 pg/mL in stage 0 to 88.2 ± 18.1 pg/mL in stage IV (*p* < 0.001 for both), with a strong correlation between IL-8 and OLGIM stage (ρ = 0.61, *p* < 0.001). The addition of OLGA/OLGIM staging to IL-8 did not result in a statistically significant improvement in discrimination compared with IL-8 alone (AUC 0.86 vs. 0.84, *p* = 0.32). Given the limited number of progression events, this finding should be interpreted cautiously and does not establish equivalence between IL-8 and OLGA/OLGIM. Nevertheless, the similar observed discrimination warrants further investigation of IL-8 as a potential non-invasive adjunctive marker associated with established histopathological risk.

### Progression to gastric cancer during 24-month follow-up

Among the 100 patients with precancerous lesions enrolled in the longitudinal cohort, 94 completed the 24-month follow-up with repeat endoscopy and histopathological evaluation. During the follow-up period, 9 patients progressed to gastric cancer, representing 9.6% of those completing follow-up and 9% of the intention-to-follow cohort. One patient died from an unrelated cause (road traffic accident) during follow-up and was censored at the time of death. Given the low competing event rate (1%), standard survival analyses were considered appropriate, and competing risk models were not required. Table 5 details the progression outcomes stratified by baseline histopathology.

The majority of progressors originated from the intestinal metaplasia group, with 7 patients progressing (14.6% of the intestinal metaplasia cohort), compared to only 2 patients from the atrophic gastritis group (4.2% of the atrophic gastritis cohort). This difference was statistically significant (*p* = 0.036), with intestinal metaplasia conferring a nearly 4-fold increased risk of progression (odds ratio 3.9, 95% CI: 1.1–13.8).

The median time to progression was 18 months (range 12–24 months). Six patients (67%) were diagnosed at the scheduled 24-month endoscopy, while 3 patients (33%) presented earlier with alarm symptoms (new-onset dyspepsia and unintentional weight loss) and were diagnosed at an interim endoscopy; all 3 originated from the intestinal metaplasia group.

Histopathological evaluation of the progressed cancers revealed that 7 (78%) were intestinal-type adenocarcinomas and 2 (22%) were diffuse-type, consistent with the expected distribution of gastric cancer subtypes arising from the intestinal metaplasia cascade. Subtype-specific statistical analysis was not performed due to the limited number of diffuse-type cases (*n* = 2). Among the intestinal-type cancers, 5 (71%) arose from patients with baseline incomplete (type III) intestinal metaplasia, while only 2 (29%) arose from those with complete (type I) intestinal metaplasia.

No cases of isolated low-grade or high-grade dysplasia were identified during follow-up. In all 9 patients who progressed to gastric cancer, invasive adenocarcinoma was the first detected neoplastic outcome. Dysplastic changes, when present, were observed only adjacent to invasive carcinoma and were not analyzed as separate outcomes.

The 12-month surveillance endoscopy provided critical insights into the temporal relationship between IL-8 dynamics and neoplastic transformation. No patient in the atrophic gastritis group and only 2 patients in the intestinal metaplasia group (4.2%) had endoscopically detectable neoplasia at this interim time point; both were confirmed to have early-stage gastric cancer (stage IA and IB) and underwent early therapeutic intervention. Critically, the remaining 7 progressors had negative 12-month endoscopies but were diagnosed with gastric cancer at the 24-month examination. Among these 7 patients, all demonstrated significant IL-8 acceleration between the 12-month and 18-month measurements (mean slope 4.2 ± 1.1 pg/mL/month), indicating that the biomarker rise preceded endoscopic visibility by a mean of 11.0 ± 3.3 months (range 6–15 months) in asymptomatic patients. All cancers detected at 24 months were early stage (I or II).

**Table 5:** Progression to Gastric Cancer During 24-Month Follow-up

Parameter	Atrophic Gastritis (*n* = 50)	Intestinal Metaplasia (*n* = 50)	Total (*N* = 100)	Test Statistic	*p*-value
Completed follow-up, n (%)	46 (92%)	48 (96%)	94 (94%)	χ² = 0.71	0.40
Progressed to GC, n (% of completed)	2 (4.3%)	7 (14.6%)	9 (9.6%)	χ² = 4.38	0.036
12-month endoscopy findings, n (% of completed)					
- Negative for neoplasia	46 (100%)	41 (85.4%)	87 (92.6%)	—	—
- Cancer detected at 12 months	0 (0%)	2 (4.2%)	2 (2.1%)	—	—
- Cancer detected at 24 months	2 (4.3%)	5 (10.4%)	7 (7.4%)	—	—
Time to progression, months, median (range)	20 (18–24)	18 (12–24)	18 (12–24)	—	—
Diagnosed at scheduled 24-mo endoscopy, n (% of progressors)	2 (100%)	4 (57%)	6 (67%)	—	—
Diagnosed earlier due to symptoms, n (% of progressors)	0 (0%)	3 (43%)	3 (33%)	—	—
Histological type at progression, n (% of progressors)					
- Intestinal type	1 (50%)	6 (86%)	7 (78%)	—	—
- Diffuse type	1 (50%)	1 (14%)	2 (22%)	—	—

Percentages for progression-related outcomes (Progressed to GC, 12-month endoscopy findings) are calculated using the number of patients who completed follow-up as the denominator (atrophic gastritis: *n* = 46; intestinal metaplasia: *n* = 48; total: *n* = 94). Percentages for timing of diagnosis and histological type are calculated using the number of progressors as the denominator (atrophic gastritis: *n* = 2; intestinal metaplasia: *n* = 7; total: *n* = 9). Completion rate calculated using enrolled patients (*n* = 50 per group) as the denominator

### Baseline IL-8 as a predictor of progression

Patients who progressed to gastric cancer during follow-up had significantly higher baseline IL-8 levels compared to those who did not progress. Among the 94 patients who completed follow-up, mean baseline IL-8 was 58.4 ± 12.7 pg/mL in progressors versus 43.6 ± 13.1 pg/mL in non-progressors (mean difference 14.8 pg/mL, 95% CI: 6.9–22.7, *p* = 0.002). This difference remained significant when analyzed separately within each histopathological group. Among intestinal metaplasia patients, progressors had a mean baseline IL-8 of 61.2 ± 11.4 pg/mL compared to 48.3 ± 12.8 pg/mL in non-progressors (mean difference 12.9 pg/mL, 95% CI: 4.8–21.0, *p* = 0.004). Among atrophic gastritis patients, the two progressors had baseline IL-8 levels of 49.1 and 56.3 pg/mL, both exceeding the group mean of 41.8 ± 11.2 pg/mL for non-progressors.

**Fig. 5:**
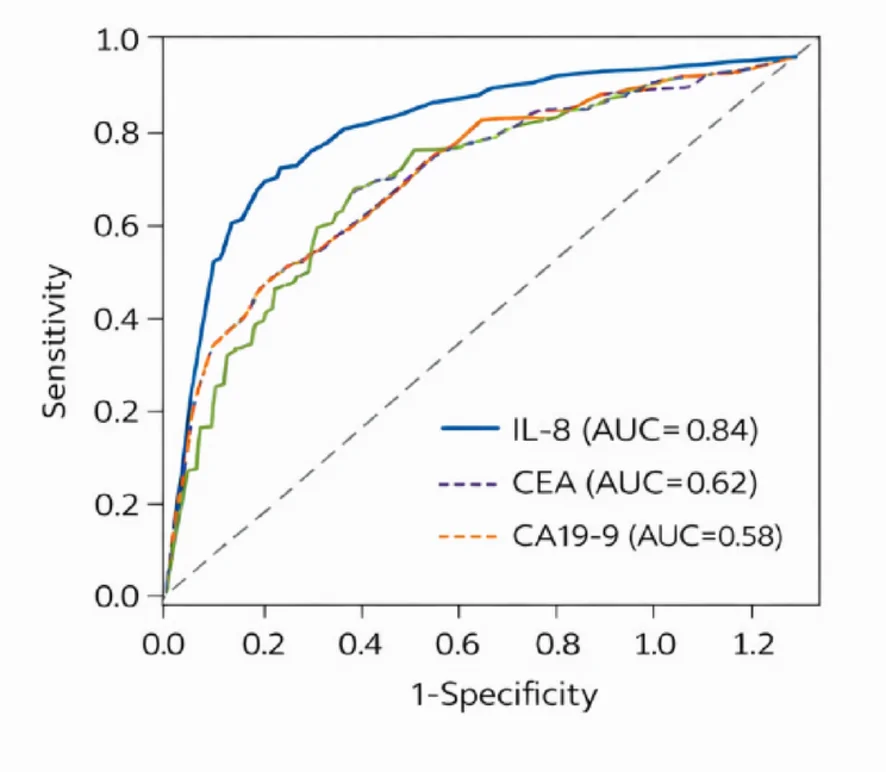
Receiver Operating Characteristic (ROC) Curves for Prediction of Progression to Gastric Cancer. ROC curves compare the discriminatory performance of baseline serum IL-8 (blue solid line), carcinoembryonic antigen (CEA; purple dashed line), and carbohydrate antigen 19 − 9 (CA19-9; orange dotted line) for predicting progression to gastric cancer within 24 months among patients with precancerous lesions (*n* = 94). The diagonal gray dashed line represents the line of no discrimination (AUC = 0.5). IL-8 demonstrated superior discriminatory ability with an AUC of 0.84 (95% CI: 0.76–0.91), which was significantly higher than that of CEA (AUC = 0.62, 95% CI: 0.51–0.73; *p* < 0.001) and CA19-9 (AUC = 0.58, 95% CI: 0.47–0.69; *p* < 0.001). These findings indicate that IL-8 significantly outperforms conventional tumor markers for identifying patients at risk of malignant progression

Receiver operating characteristic curve analysis was performed to evaluate the ability of baseline IL-8 to predict progression to gastric cancer. The area under the curve was 0.84 (95% CI: 0.76–0.91), indicating promising preliminary discriminatory performance **(**Fig. 5; Table 6**)**. This significantly exceeded the AUC for carcinoembryonic antigen (0.62, 95% CI: 0.51–0.73, p for comparison = 0.002) and carbohydrate antigen 19 − 9 (0.58, 95% CI: 0.47–0.69, *p* for comparison < 0.001).

The optimal cut-off value for baseline IL-8, determined by maximizing the Youden index, was 52.3 pg/mL. At this threshold, baseline IL-8 was associated with progression with high sensitivity (88.9%) and excellent negative predictive value (98.5%), as detailed in Table 6.

When the analysis was stratified by histopathological group, the predictive performance remained robust. Among intestinal metaplasia patients, the AUC for baseline IL-8 was 0.86 (95% CI: 0.77–0.94), with a sensitivity of 85.7% and a specificity of 79.5% at the 52.3 pg/mL cut-off. Among atrophic gastritis patients, the AUC was 0.79 (95% CI: 0.64–0.94), though the small number of progressors in this group limits precision.

To assess internal validity and quantify optimism in our predictive model, we performed bootstrap resampling with 1000 iterations using the 0.632 + estimator. The optimism-corrected AUC for baseline IL-8 was 0.82 (95% CI: 0.74–0.89), indicating minimal overfitting and stable performance. The calibration slope, estimated from bootstrap validation, was 0.94 (95% CI: 0.87–1.01), suggesting excellent agreement between predicted and observed probabilities. For the multivariable Firth logistic regression model presented in Table 7, bootstrap validation yielded an optimism-corrected C-statistic of 0.85 (95% CI: 0.77–0.92).

To assess the stability of the optimal cut-off value, we performed bootstrap resampling with 1000 iterations, recalculating the Youden index-derived cut-off in each bootstrap sample. The median bootstrap cut-off was 52.1 pg/mL (95% CI: 48.7–55.9 pg/mL), and the cut-off of 52.3 pg/mL was within the optimal range in 94% of bootstrap samples.

The positive likelihood ratio of 4.04 indicates that a positive test (IL-8 > 52.3 pg/mL) is approximately four times more likely to occur in a patient who will progress than in one who will not, while the negative likelihood ratio of 0.14 indicates that a negative test substantially reduces the probability of progression. The overall accuracy was 78.7%, and the Youden index was 0.669.

**Fig. 6:**
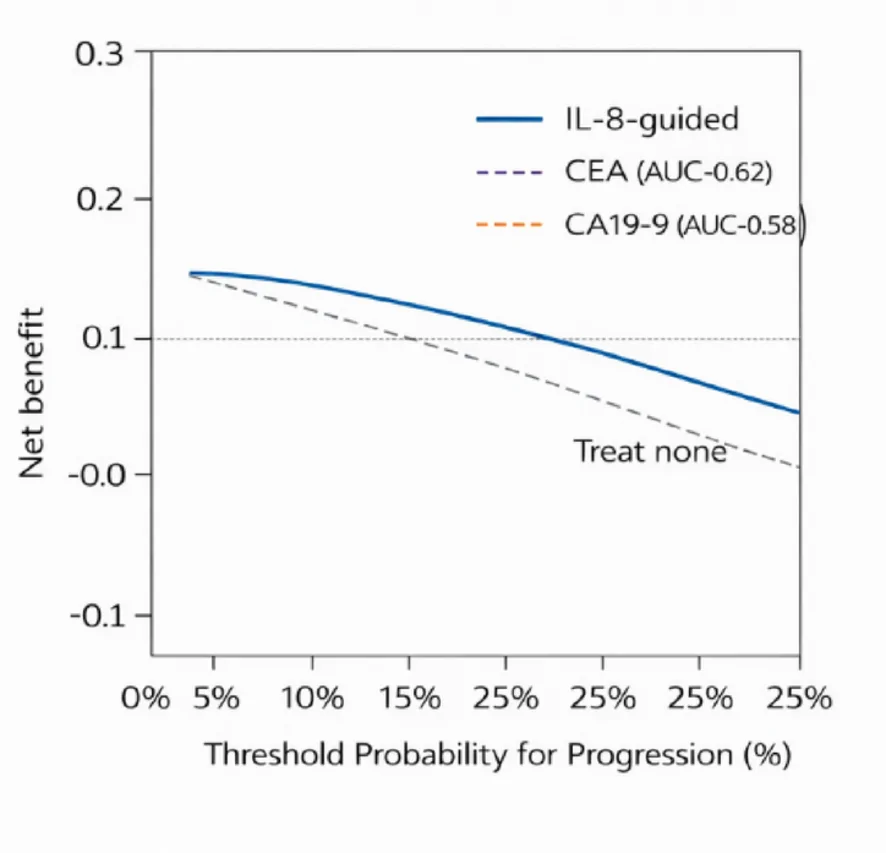
Decision Curve Analysis for IL-8-Guided Risk Stratification. Decision curve analysis evaluating the clinical utility of using baseline IL-8 > 52.3 pg/mL to guide surveillance decisions. The y-axis represents net benefit, and the x-axis represents threshold probability for progression to gastric cancer. The blue line shows the net benefit of the IL-8-guided strategy. The horizontal gray line represents the strategy of performing surveillance in no patients (treat none), and the dashed purple line represents the strategy of performing surveillance in all patients (treat all). The IL-8-guided strategy demonstrates positive net benefit across threshold probabilities from 5% to 25%, indicating that using IL-8 to select patients for intensified surveillance would yield better clinical outcomes than strategies of performing surveillance in all or no patients across a clinically relevant range of risk thresholds. These findings support the potential clinical utility of IL-8-guided risk stratification

To evaluate the potential clinical utility of IL-8-guided risk stratification, we performed decision curve analysis (Fig. 6)**.** Using baseline IL-8 > 52.3 pg/mL to guide surveillance decisions provided positive net clinical benefit across threshold probabilities ranging from 5% to 25%.

To address the concern of reverse causality inherent in post-baseline trajectory analysis, we performed Cox proportional hazards regression using baseline IL-8 only (measured at enrollment before any cancer diagnosis). Baseline IL-8 > 52.3 pg/mL was significantly associated with an increased hazard of progression to gastric cancer (unadjusted HR: 8.42, 95% CI: 3.21–22.08, *p* < 0.001). After adjustment for histopathological group (intestinal metaplasia vs. atrophic gastritis), baseline IL-8 > 52.3 pg/mL remained independently associated with progression (adjusted HR: 6.94, 95% CI: 2.58–18.67, *p* < 0.001).

**Fig. 7:**
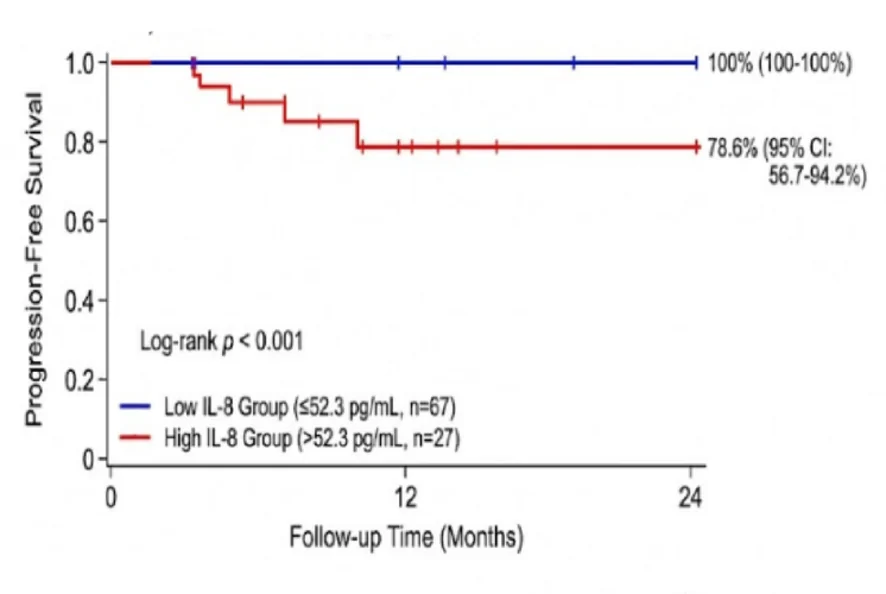
Kaplan–Meier curves for progression to gastric cancer stratified by baseline IL-8 level. Patients with precancerous lesions (atrophic gastritis and intestinal metaplasia, *n* = 94) were stratified using the optimal baseline IL-8 cut-off of 52.3 pg/mL derived from ROC analysis. The high IL-8 group (> 52.3 pg/mL, red line, *n* = 27) demonstrated significantly lower progression-free survival compared to the low IL-8 group (≤ 52.3 pg/mL, blue line, *n* = 67). Log-rank *p* < 0.001. Shaded areas represent 95% confidence intervals. Numbers at risk are shown below the x-axis. Tick marks indicate censored patients

Kaplan–Meier analysis demonstrated significantly lower progression-free survival among patients with baseline IL-8 > 52.3 pg/mL compared to those below this threshold (log-rank *p* < 0.001). The median progression-free survival was not reached in either group due to the limited number of events and the 24-month follow-up duration, but the curves separated early (by 12 months) and remained divergent throughout follow-up **(**Fig. 7**)**. The 24-month progression-free survival was 100% (95% CI: 100–100%) in the low IL-8 group (≤ 52.3 pg/mL) and 78.6% (95% CI: 56.7–94.2%) in the high IL-8 group (> 52.3 pg/mL). Sensitivity analysis for unmeasured confounding yielded an E-value of approximately 13.3 for the observed adjusted hazard ratio of 6.94 and 4.6 for the confidence limit closest to the null (HR 2.58). Thus, substantial unmeasured confounding would be required to fully explain the observed association.

**Table 6:** Predictive Performance of Baseline IL-8 for Progression to Gastric Cancer

Performance Metric	Value (95% Confidence Interval)
AUC (95% CI)	0.84 (0.76–0.91)
Optimal cut-off	52.3 pg/mL
Sensitivity	88.9% (51.8–99.7%)
Specificity	78.0% (68.1–85.9%)
Positive Predictive Value	29.6% (18.2–44.2%)
Negative Predictive Value	98.5% (91.7–99.9%)
Positive Likelihood Ratio	4.04 (2.67–6.11)
Negative Likelihood Ratio	0.14 (0.02–0.93)
Accuracy	78.7% (69.5–86.2%)
Youden Index	0.669

AUC: area under the receiver operating characteristic curve

### Factors independently associated with progression: multivariable analysis

Univariate logistic regression analysis identified several factors associated with progression to gastric cancer, as detailed in Table 7. Baseline IL-8 (OR per 10 pg/mL increase: 2.14, 95% CI: 1.42–3.23, *p* < 0.001) and intestinal metaplasia (OR 3.92, 95% CI: 1.08–14.21, *p* = 0.038) were significantly associated with progression in univariate analysis, while age (*p* = 0.06) and hemoglobin (*p* = 0.08) showed trends toward association. Conventional tumor markers, carcinoembryonic antigen and carbohydrate antigen 19 − 9, were not significantly associated with progression (*p* = 0.24 and *p* = 0.31, respectively).

Multivariable logistic regression analysis was performed using backward stepwise selection based on Akaike Information Criterion, including all variables with *p* < 0.10 in univariate analysis. The final model retained baseline IL-8 and histopathological group as factors independently associated with progression **(**Table 7**)**. Baseline IL-8 remained highly significant, with each 10 pg/mL increase associated with a 2.31-fold increase in the odds of progression (adjusted OR 2.31 per 10 pg/mL, 95% CI: 1.48–3.61, *p* < 0.001). Intestinal metaplasia independently conferred a 4.2-fold increased risk compared to atrophic gastritis (adjusted OR 4.22, 95% CI: 1.31–13.61, *p* = 0.016). Age and hemoglobin were not retained in the final model, as their inclusion did not improve model fit.

The final multivariable model demonstrated excellent calibration (Hosmer-Lemeshow χ² = 6.84, *p* = 0.45) and discrimination (C-statistic = 0.87, 95% CI: 0.79–0.95). The addition of IL-8 to a model containing only the histopathological group significantly improved risk prediction, with a net reclassification improvement of 0.42 (95% CI: 0.21–0.63, *p* < 0.001) and an integrated discrimination improvement of 0.18 (95% CI: 0.09–0.27, *p* < 0.001), confirming the incremental value of IL-8 measurement.

**Table 7:** Univariate and Multivariable Logistic Regression Analysis for Prediction of Progression

Variable	Univariate Analysis		Multivariable Analysis	
	OR (95% CI)	*p*-value	aOR (95% CI)	*p*-value
IL-8 (per 10 pg/mL)	2.14 (1.42–3.23)	< 0.001	2.31 (1.48–3.61)	< 0.001
Histopathological group				
- Atrophic gastritis	Reference	—	Reference	—
- Intestinal metaplasia	3.92 (1.08–14.21)	0.038	4.22 (1.31–13.61)	0.016
Age (per 10 years)	1.58 (0.98–2.55)	0.06	Not retained	—
Sex (male vs. female)	1.24 (0.45–3.42)	0.68	—	—
Hemoglobin (per 1 g/dL decrease)	1.45 (0.96–2.19)	0.08	Not retained	—
CEA (per 1 ng/mL)	1.03 (0.98–1.08)	0.24	—	—
CA19-9 (per 10 U/mL)	1.02 (0.98–1.06)	0.31	—	—
Smoking (current vs. never)	1.56 (0.56–4.35)	0.39	—	—

Multivariable model performed using backward stepwise selection based on Akaike Information Criterion. Hosmer-Lemeshow goodness-of-fit *p* = 0.45. C-statistic = 0.87 (95% CI: 0.79–0.95). OR: odds ratio; aOR: adjusted odds ratio; CI: confidence interval; CEA: carcinoembryonic antigen; CA19-9: carbohydrate antigen 19 − 9

### Longitudinal IL-8 trajectories: identification of high-risk patterns

Having established that IL-8 levels increase progressively across the carcinogenesis spectrum and are elevated even in *H. pylori*-negative cancers, we next evaluated whether baseline and serial IL-8 measurements could predict progression to cancer specifically in patients with *H. pylori*-associated precancerous lesions, the population for whom non-invasive risk stratification would have the greatest clinical impact. Critically, all patients underwent protocol-mandated surveillance endoscopy at 12 months in addition to the final 24-month examination, enabling precise determination of the temporal relationship between IL-8 dynamics and neoplastic transformation.

Linear mixed-effects modeling of serial IL-8 measurements over the 24-month follow-up period revealed fundamentally different trajectory patterns between patients who progressed to cancer and those who did not. The model included fixed effects for time, progression status, and their interaction, with random intercepts and random slopes for time at the patient level. The time-by-progression interaction was highly significant (F = 24.6, *p* < 0.001).

Among non-progressors, IL-8 levels remained relatively stable throughout follow-up, with a mean slope of 0.8 pg/mL per month (95% CI: 0.4–1.2 pg/mL/month), representing a modest increase of approximately 9.6 pg/mL over the entire 24-month period. In contrast, progressors demonstrated a steeply accelerating trajectory, with a mean slope of 3.2 pg/mL per month (95% CI: 2.4-4.0 pg/mL/month), corresponding to an average increase of 38.4 pg/mL over 24 months. The difference in slopes between groups was 2.4 pg/mL/month (95% CI: 1.6–3.2 pg/mL/month, *p* < 0.001).

Importantly, the divergence in trajectories became apparent beginning at 12 months of follow-up. At 6 months, IL-8 levels in progressors (mean 62.1 ± 14.3 pg/mL) were only modestly higher than in non-progressors (mean 51.2 ± 13.8 pg/mL), with considerable overlap between groups. By 12 months, the separation became more pronounced (progressors: 71.4 ± 16.2 pg/mL; non-progressors: 54.6 ± 14.1 pg/mL), and by 18 and 24 months, the trajectories were clearly distinct, with progressors reaching mean levels of 84.3 ± 18.7 pg/mL at 18 months and 96.8 ± 21.4 pg/mL at 24 months, compared to 57.8 ± 15.2 pg/mL and 61.4 ± 16.8 pg/mL in non-progressors at the same time points.

Using group-based trajectory modeling performed blinded to outcome status, we identified three distinct trajectory patterns detailed in Table 8. In this exploratory analysis, the first pattern, designated Stable-Low, comprised 42 patients (45% of the cohort) and was characterized by consistently low IL-8 levels throughout follow-up, with baseline levels of 32.4 ± 6.8 pg/mL and 24-month levels of 35.1 ± 7.2 pg/mL, demonstrating minimal change over time (slope 0.2 pg/mL/month). The average posterior probability for this group was 0.91, indicating excellent classification certainty. The second pattern, designated Moderate-Rising, included 49 patients (52% of the cohort) and was characterized by moderately elevated baseline IL-8 levels of 48.7 ± 8.3 pg/mL with gradual increase over time (slope 0.9 pg/mL/month), reaching 62.3 ± 10.4 pg/mL at 24 months. The average posterior probability for this group was 0.88.

**Fig. 8:**
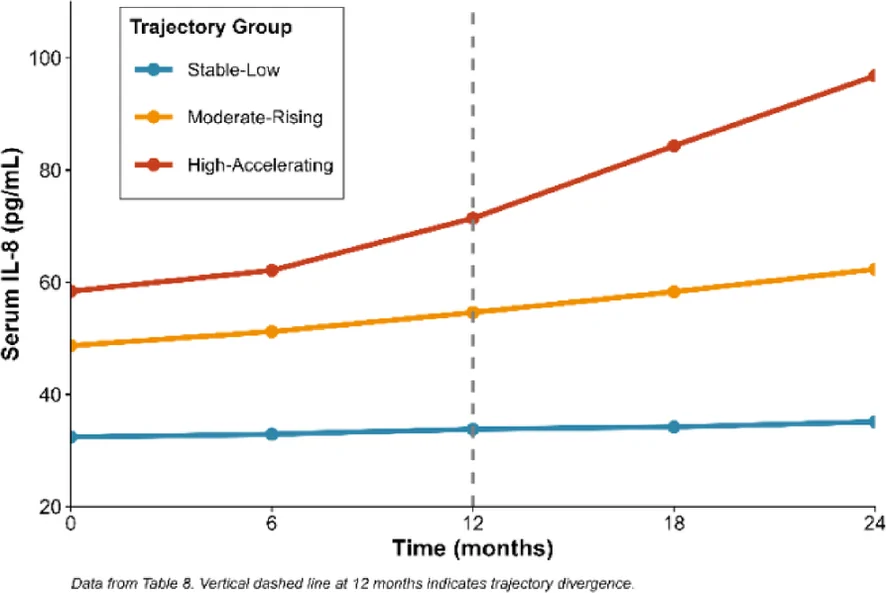
Longitudinal IL-8 Trajectories Identified by Group-Based Trajectory Modeling. Line graph showing the three distinct IL-8 trajectory patterns over 24 months of follow-up among patients with precancerous lesions (*n* = 94). The Stable-Low trajectory (blue line, *n* = 42) is characterized by consistently low IL-8 levels throughout follow-up (baseline: 32.4 ± 6.8 pg/mL; 24-month: 35.1 ± 7.2 pg/mL). The Moderate-Rising trajectory (orange line, *n* = 49) shows moderately elevated baseline levels (48.7 ± 8.3 pg/mL) with gradual increase over time, reaching 62.3 ± 10.4 pg/mL at 24 months. The High-Accelerating trajectory (red line, *n* = 9) demonstrates high baseline levels (58.4 ± 9.1 pg/mL) with striking acceleration beginning at 12 months (slope pre-12: 1.1 pg/mL/month; slope post-12: 4.8 pg/mL/month), resulting in 24-month levels of 96.8 ± 15.2 pg/mL. The vertical dashed line at 12 months highlights the point of trajectory divergence. All 9 patients who progressed to gastric cancer were classified into the High-Accelerating trajectory group. Shaded areas represent 95% confidence intervals (data from Table 8)

The third pattern, designated High-Accelerating, included only 9 patients (10% of the cohort) but demonstrated the most clinically significant features, with high baseline IL-8 levels of 58.4 ± 9.1 pg/mL and a striking acceleration beginning at 12 months, clearly visualized in Fig. 8. Before 12 months, the slope in this group was 1.1 pg/mL/month, but after 12 months, the slope increased dramatically to 4.8 pg/mL/month, resulting in 24-month IL-8 levels of 96.8 ± 15.2 pg/mL. The average posterior probability for this group was 0.94.

Remarkably, all 9 patients who progressed to gastric cancer were classified into the High-Accelerating trajectory group, representing 100% of progressors in our cohort and 100% of patients in this trajectory class. However, this exploratory finding should be interpreted with caution, as post-baseline IL-8 measurements may have been obtained after some patients had already developed subclinical malignancy. Therefore, the trajectory analysis is presented as hypothesis-generating, and the primary predictive evidence comes from baseline IL-8 analysis (Cox regression and Kaplan–Meier, Fig. 7), which avoids reverse causality. This perfect separation, while striking, requires validation in larger, independent cohorts to determine whether it reflects a true biological phenomenon or is influenced by the limited number of events.

To assess the stability of the trajectory solution and exclude overfitting, we performed sensitivity analyses using three approaches. First, we conducted leave-one-out cross-validation, iteratively refitting the trajectory model excluding each progressor in turn; the three-class solution, assigning all remaining progressors to the High-Accelerating group, was preserved in 100% of iterations. Second, we performed 10-fold cross-validation, which yielded identical trajectory assignments in 93 of 94 patients (99%). Third, we refitted the model using a random 70% derivation sample (*n* = 66) and validated the solution in the remaining 30% (*n* = 28); the three-class structure and perfect separation of progressors were reproduced in both samples. These analyses, together with the bootstrap stability confidence intervals shown in Table 8.

The temporal pattern of the High-Accelerating trajectory is particularly noteworthy from a clinical perspective. Among the 9 progressors, the acceleration point, defined as the time at which the slope increased by more than 2 pg/mL/month compared to baseline, occurred at a median of 12 months (range 9–15 months). This acceleration preceded the actual cancer diagnosis by a median of 6 months (range 3–12 months), identifying a potential therapeutic window during which patients are at high risk but have not yet developed endoscopically detectable malignancy. While the perfect separation of progressors into the High-Accelerating group in our cohort suggests excellent discriminatory performance, the confidence intervals around these predictive values remain wide (PPV 95% CI: 66–100%).

**Table 8:** Characteristics of IL-8 Trajectory Groups

Characteristic	Stable-Low (*n* = 42)	Moderate-Rising (*n* = 49)	High-Accelerating (*n* = 9)	Test Statistic	*p*-value
IL-8 levels, pg/mL, mean ± SD					
- Baseline	32.4 ± 6.8	48.7 ± 8.3	58.4 ± 9.1	F = 52.6	< 0.001
− 6 months	32.9 ± 6.9	51.2 ± 8.7	62.1 ± 10.4	F = 64.2	< 0.001
− 12 months	33.8 ± 7.1	54.6 ± 9.2	71.4 ± 12.3	F = 78.4	< 0.001
− 18 months	34.2 ± 7.0	58.3 ± 9.8	84.3 ± 14.2	F = 94.6	< 0.001
− 24 months	35.1 ± 7.2	62.3 ± 10.4	96.8 ± 15.2	F = 112.7	< 0.001
Trajectory slopes, pg/mL/month (95% CI)					
- Overall slope	0.2 (0.1–0.3)	0.9 (0.7–1.1)	3.2 (2.6–3.8)	F = 86.4*	< 0.001*
- Pre-12-month slope	0.2 (0.1–0.3)	0.8 (0.6-1.0)	1.1 (0.5–1.7)	F = 12.4*	0.002*
- Post-12 month slope	0.2 (0.1–0.3)	1.0 (0.8–1.2)	4.8 (4.1–5.5)	F = 124.8*	< 0.001*
- Slope change (post-12 vs. pre-12)	0.0 (-0.1 to 0.1)	0.2 (0.1–0.3)	3.7 (3.0-4.4)	F = 98.2*	< 0.001*
Progression outcomes					
- Progression to cancer, n (%)	0 (0%)	0 (0%)	9 (100%)	χ² = 94.0	< 0.001
− 12-month endoscopy negative, n (%)	42 (100%)	49 (100%)	7 (78%)**	χ² = 28.6	< 0.001
- Lead time (months), mean ± SD	—	—	8.0 ± 5.2	—	—
- Lead time in asymptomatic patients***, mean ± SD	—	—	11.0 ± 3.3	—	—
Histopathological characteristics					
- Histopathological group				χ² = 28.4	< 0.001
- Atrophic gastritis, n (%)	28 (67%)	18 (37%)	2 (22%)		
- Intestinal metaplasia, n (%)	14 (33%)	31 (63%)	7 (78%)		
- Incomplete metaplasia (type III), n (% of IM)	4 (29%)	13 (42%)	5 (71%)	χ² = 6.2	0.045
Model fit indices					
- Average posterior probability	0.91	0.88	0.94	—	—
- Entropy (overall model)		0.86		—	—
- Bootstrap stability (95% CI for class size)	38–46	44–54	7–11	—	—

SD: standard deviation; CI: confidence interval; IM: intestinal metaplasia. *: F-statistic and p-value for the overall test of slope differences between trajectory groups. **: Two patients in the High-Accelerating group had cancer detected at 12-month endoscopy. ***: Asymptomatic patients with negative 12-month endoscopy (*n* = 6). Lead time calculated as months between acceleration point (> 2 pg/mL/month increase) and cancer diagnosis. P-values from ANOVA for continuous variables and chi-square test for categorical variables. Trajectory groups were identified using group-based trajectory modeling with Bayesian Information Criterion optimization. Bayesian Information Criterion values for trajectory models: 2-class: 2846; 3-class: 2712; 4-class: 2708; 5-class: 2705. The 3-class solution was selected based on clinical interpretability and parsimony despite a similar BIC to 4-class models. Bootstrap validation performed with 500 replications.Positive predictive value of High-Accelerating trajectory: 100% (95% CI: 66–100%); negative predictive value of combined Stable-Low/Moderate-Rising trajectories: 100% (95% CI: 96–100%). The multiple statistical comparisons presented in Table 8 were not adjusted for multiplicity, reflecting the exploratory objective of trajectory phenotyping

### Impact of *H. pylori* eradication on IL-8 trajectories

Following baseline endoscopy, all 100 patients with precancerous lesions received standardized eradication therapy in accordance with Maastricht VI guidelines. Successful eradication, confirmed by a negative 13 C-urea breath test at least 4 weeks post-treatment, was achieved in 86 patients (86%), while 14 patients (14%) had persistent infection and received second-line therapy per local protocols. Of the 14 patients with persistent infection after first-line therapy, 12 (85.7%) achieved successful eradication following second-line treatment, while 2 patients (1.4% of the original cohort) remained *H. pylori*-positive at the end of follow-up. Both patients who remained persistently infected had baseline intestinal metaplasia (Group II); neither progressed to gastric cancer during the 24-month follow-up period. All 14 patients with persistent infection remained classified as *H. pylori*-positive throughout follow-up and were included in all analyses according to their baseline infection status. All patients, regardless of eradication outcome, remained classified in their original groups (I or II) based on baseline infection status for all longitudinal analyses, consistent with the intention-to-diagnose principle. Table 9 summarizes the impact of eradication status on IL-8 dynamics.

To assess whether eradication influenced subsequent IL-8 dynamics and potentially confounded trajectory analyses, we compared changes in IL-8 before and after treatment in patients who were successfully eradicated versus those with persistent infection. Among successfully treated patients, mean IL-8 levels decreased significantly from baseline to 6 months (mean change − 3.3 pg/mL, 95% CI: -5.1 to -1.5, *p* = 0.001), while no significant change was observed in patients with persistent infection (mean change − 0.5 pg/mL, 95% CI: -2.8 to 1.8, *p* = 0.64). The difference in early IL-8 response between groups was highly significant (*p* = 0.001).

**Post-eradication IL-8 levels in successfully treated patients compared to never-infected individuals**. To address whether IL-8 levels normalize after successful eradication, we compared 6-month IL-8 measurements (post-eradication) from successfully treated patients in Groups I and II (*n* = 86, mean 45.3 ± 12.8 pg/mL) with baseline IL-8 from never-infected healthy controls (Group V, *n* = 50, mean 9.7 ± 3.2 pg/mL) and never-infected gastric cancer patients (Group IV, *n* = 25, mean 98.6 ± 22.1 pg/mL). Post-eradication IL-8 in successfully treated patients was significantly higher than in healthy controls (mean difference 35.6 pg/mL, 95% CI: 31.2–40.0, *p* < 0.001) but significantly lower than in *H. pylori*-negative gastric cancer patients (mean difference − 53.3 pg/mL, 95% CI: -62.1 to -44.5, *p* < 0.001). When stratified by eventual outcome, successfully eradicated non-progressors (*n* = 78) had significantly lower 6-month IL-8 levels (42.8 ± 11.4 pg/mL) compared to successfully eradicated progressors (*n* = 8, 68.2 ± 14.6 pg/mL, mean difference 25.4 pg/mL, 95% CI: 15.8–35.0, *p* < 0.001). Notably, 6-month IL-8 in eradicated non-progressors remained elevated compared to healthy controls (42.8 vs. 9.7 pg/mL, *p* < 0.001), suggesting that even after successful eradication, patients with a history of precancerous lesions maintain higher baseline inflammatory markers than never-infected individuals.

However, when examining long-term trajectories from 6 to 24 months, the period during which trajectory divergence became apparent, there were no significant differences in IL-8 slopes between successfully eradicated patients and those with persistent infection (0.9 ± 0.4 vs. 1.0 ± 0.5 pg/mL/month, *p* = 0.34). Critically, among the 9 patients who progressed to cancer, 8 (89%) had successful eradication, and all 9 were classified in the High-Accelerating trajectory group regardless of eradication status.

When stratified by eventual outcome, successfully eradicated non-progressors (*n* = 78) showed a significant early IL-8 decrease (mean change − 3.5 ± 2.1 pg/mL, *p* < 0.001), while the 8 eradicated progressors showed no significant early change (mean change − 0.8 ± 2.4 pg/mL, *p* = 0.48).

To formally assess whether eradication status confounded our longitudinal analyses, we performed a sensitivity analysis comparing IL-8 trajectories before and after eradication in successfully treated patients versus those with persistent infection. No significant differences in long-term IL-8 trajectories were observed between these groups (*p* = 0.34).

The proportion of patients who progressed to cancer did not differ significantly between those who achieved successful eradication (8/86, 9.3%) and those with persistent infection (1/14, 7.1%; *p* = 0.79). The single progressor in the persistent infection group was among the 12 patients who achieved eradication after second-line therapy, not among the 2 who remained persistently infected.

The High-Accelerating phenotype was observed across all eradication outcome categories: patients who achieved eradication after first-line therapy (*n* = 8), after second-line therapy (*n* = 1), and among persistently infected patients (the phenotype was present, though none progressed in this subgroup).

All 14 patients with persistent infection remained classified as *H. pylori*-positive throughout follow-up and were included in all analyses according to their baseline infection status. Of these, 12 (85.7%) achieved successful eradication after second-line therapy, while 2 patients (1.4% of the original cohort) remained H. pylori-positive at the end of follow-up. No significant differences in long-term IL-8 trajectories were observed between successfully eradicated and persistently infected patients (*p* = 0.34), indicating that eradication status did not confound trajectory analyses.

**Table 9:** Impact of *H. pylori* Eradication on IL-8 Dynamics

Parameter	Eradicated (*n* = 86)	Failed first-line eradication (*n* = 14)	*p*-value
Baseline IL-8, pg/mL	48.6 ± 13.2	49.2 ± 14.1	0.87
IL-8 at 6 months, pg/mL	45.3 ± 12.8	48.7 ± 13.9	0.36
Change baseline to 6 months, pg/mL	-3.3 (-5.1 to -1.5)	-0.5 (-2.8 to 1.8)	0.001
Long-term slope (6–24 mo), pg/mL/month	0.9 ± 0.4	1.0 ± 0.5	0.34
Progressed to cancer, n (%)	8 (9.3%)	1 (7.1%)	0.79

All 14 patients with persistent infection remained classified as H. pylori-positive throughout follow-up and were included in all analyses according to their baseline infection status. Of these, 12 (85.7%) achieved successful eradication after second-line therapy, while 2 patients (1.4% of the original cohort) remained H. pylori-positive at the end of follow-up. No significant differences in long-term IL-8 trajectories were observed between successfully eradicated and persistently infected patients (p = 0.34), indicating that eradication status did not confound trajectory analyses. The column label ‘Failed first-line eradication’ refers to the status after first-line therapy; 12 of these 14 patients achieved eradication after second-line therapy

### Time-dependent predictive performance of serial IL-8 measurements

Time-dependent receiver operating characteristic analysis was performed to assess how the predictive accuracy of IL-8 evolved over the follow-up period. Cumulative/dynamic AUCs were calculated for IL-8 measured at baseline, 6, 12, and 18 months, each predicting progression occurring within the subsequent follow-up interval. Table 10 summarizes these time-dependent performance metrics.

The predictive accuracy of IL-8 increased progressively over time. The AUC for baseline IL-8 predicting progression over the entire 24-month period was 0.84 (95% CI: 0.76–0.91). For IL-8 measured at 6 months predicting progression over the remaining 18 months, the AUC increased to 0.87 (95% CI: 0.80–0.94). By 12 months, the AUC reached 0.91 (95% CI: 0.85–0.97), and at 18 months, the AUC was 0.94 (95% CI: 0.89–0.99).

The optimal cut-off for risk stratification also evolved over time, reflecting the rising IL-8 levels in progressors. As shown in Table 10, the cut-off increased from 52.3 pg/mL at baseline to 72.8 pg/mL at 18 months, while sensitivity remained stable at 88.9%. Notably, specificity improved from 78.0% at baseline to 92.5% at 18 months, and positive predictive value nearly doubled from 29.6% to 57.1%, indicating that later measurements provide superior discrimination. The negative predictive value remained exceptionally high throughout (98.5–99.0%) across all time points.

**Table 10:** Time-Dependent Predictive Performance of Serial IL-8 Measurements

Time Point	AUC (95% CI)	Optimal Cut-off (pg/mL)	Sensitivity (%)	Specificity (%)	PPV (%)	NPV (%)
Baseline	0.84 (0.76–0.91)	52.3	88.9	78.0	29.6	98.5
6 months	0.87 (0.80–0.94)	58.7	88.9	84.9	38.1	98.7
12 months	0.91 (0.85–0.97)	64.2	88.9	89.2	47.1	98.8
18 months	0.94 (0.89–0.99)	72.8	88.9	92.5	57.1	99.0

AUC: area under the receiver operating characteristic curve; CI: confidence interval; PPV: positive predictive value; NPV: negative predictive value. Time-dependent AUCs calculated using cumulative/dynamic methodology with Kaplan-Meier estimator. All measurements were performed on the 94 patients who completed follow-up

### Subgroup and sensitivity analyses

Pre-specified subgroup analyses were performed to assess whether the predictive value of IL-8 varied across key patient populations. Table 11 summarizes the AUCs for baseline IL-8 predicting progression across all predefined subgroups. The predictive performance remained consistent across sexes (AUC 0.85 in males, 0.83 in females), age groups (0.86 in patients < 60 years, 0.82 in those ≥ 60 years), and smoking status (0.84 in non-smokers, 0.85 in current smokers). There were no significant interactions between IL-8 and any of these variables (all *p* for interaction > 0.20).

Analysis by center revealed consistent IL-8 predictive performance across all five participating sites, with AUCs ranging from 0.81 to 0.88, as detailed in Table 11. To formally assess potential inter-center variability, we included center as a random effect in mixed models and tested for center-by-IL-8 interactions. No significant center effects were detected (*p* > 0.20 for all comparisons), and the intraclass correlation coefficient for center was 0.02, indicating that less than 2% of the variance in IL-8 was attributable to site-specific factors.

Several sensitivity analyses were performed to assess the robustness of our findings to different analytical assumptions. Complete case analysis excluding the 6 patients lost to follow-up yielded nearly identical results to the primary analysis, with an AUC of 0.84 (95% CI: 0.76–0.91) for baseline IL-8. Multiple imputation for missing IL-8 values using chained equations with 20 imputed datasets produced a pooled AUC of 0.83 (95% CI: 0.75–0.91), consistent with the primary analysis. Exclusion of patients with protocol violations or inadequate biopsy sampling (*n* = 7) did not materially change the results. Analysis using alternative IL-8 cut-off values within 10% of the optimal cut-off (47.1–57.6 pg/mL) maintained good predictive performance, with sensitivities ranging from 77.8% to 100% and specificities from 71.0% to 84.9%.

**Table 11:** Subgroup and Sensitivity Analyses for IL-8 Predictive Performance

Analysis	*n*	AUC (95% CI)	*p*-value*
Primary analysis	94	0.84 (0.76–0.91)	Reference
By sex			
- Male	55	0.85 (0.76–0.94)	0.76
- Female	39	0.83 (0.71–0.95)	0.82
By age			
- <60 years	51	0.86 (0.78–0.94)	0.68
- ≥60 years	43	0.82 (0.71–0.93)	0.71
By smoking status			
- Non-smoker	58	0.84 (0.74–0.94)	0.94
- Current smoker	36	0.85 (0.73–0.97)	0.88
By histopathological group			
- Atrophic gastritis	46	0.79 (0.64–0.94)	0.42
- Intestinal metaplasia	48	0.86 (0.77–0.94)	0.58
By center			
- Banha	22	0.86 (0.75–0.97)	0.72
- Shebin Elkom	28	0.83 (0.71–0.95)	0.84
- Damnhour	24	0.81 (0.68–0.94)	0.68
- Assuit	12	0.88 (0.76-1.00)	0.58
- Suez	8	0.85 (0.70-1.00)	0.91
Sensitivity analyses			
- Complete case analysis	94	0.84 (0.76–0.91)	—
- Multiple imputation (20 datasets)	100	0.83 (0.75–0.91)	—
- Excluding protocol violations	87	0.85 (0.77–0.93)	—

*P-values for comparison with primary analysis using the DeLong test for AUC comparisons. CI: confidence interval; AUC: area under the receiver operating characteristic curve

### Risk stratification algorithm for clinical implementation

Based on the trajectory analysis, we developed an exploratory IL-8-based risk stratification framework to illustrate how longitudinal biomarker measurements may contribute to future risk assessment in patients with histologically confirmed *H. pylori*-associated precancerous gastric lesions. The proposed framework is intended solely for hypothesis generation and should not be interpreted as a clinical surveillance algorithm.

As summarized in Table 12, patients were categorized according to baseline and 12-month serum IL-8 concentrations. During the 24-month follow-up, no progression events were observed among patients classified as low risk (0/42) or intermediate risk (0/49), whereas all nine progression events occurred within the high-risk category (9/9). These findings suggest that serial IL-8 measurements may identify distinct patterns of malignant progression; however, the small number of progression events, particularly within the high-risk group, limits the precision and generalizability of these observations.

The proposed framework should be interpreted as complementary to, rather than a replacement for, established risk assessment based on endoscopic and histopathological findings. It was not designed for population screening but for patients with confirmed precancerous gastric lesions who are already undergoing surveillance. Furthermore, the present model does not incorporate several established risk modifiers, including first-degree family history of gastric cancer, OLGA/OLGIM stage, or the extent of intestinal metaplasia, which should be integrated into future prediction models.

When applied retrospectively to our cohort, the framework identified all observed progression events while classifying 42% of non-progressors as low risk. These findings provide preliminary evidence supporting the potential value of IL-8 for risk stratification but should be regarded as exploratory and hypothesis-generating. Larger prospective studies with longer follow-up are required to validate the model, integrate established clinical risk factors, and determine whether IL-8-guided surveillance strategies improve patient outcomes or safely modify surveillance intervals.

**Table 12:** Proposed Risk Stratification Algorithm Based on IL-8 Measurements

Risk Category	Criteria	Observed Progression (24 months)	Interpretation (Exploratory)	Research Implication
Low	Baseline IL-8 < 40 pg/mL AND 12-month IL-8 < 45 pg/mL	0% (0/42)	No progression observed during 24 months	Longer surveillance intervals should be evaluated in future validation studies
Intermediate	Baseline IL-8 40-52.3 pg/mL OR 12-month IL-8 45–64 pg/mL (not meeting high-risk criteria)	0% (0/49)	Intermediate observed risk	Further validation required before modifying surveillance
High	Baseline IL-8 > 52.3 pg/mL AND 12-month IL-8 > 64 pg/mL, OR 12-month increase > 50% from baseline	100% (9/9)	Highest observed risk (*n* = 9 events)	May justify evaluation of intensified surveillance in future prospective studies

This algorithm is exploratory and based on IL-8 measurements alone. It does not replace established risk factors (e.g., family history, OLGA/OLGIM staging) and requires validation in independent cohorts before clinical application. The observed findings are hypothesis-generating and intended to guide future research, not current clinical practice

## Discussion

This prospective multicenter cohort study provides the first longitudinal evidence that serial serum IL-8 measurements may be associated with risk stratification of malignant progression in patients with *H. pylori-*associated precancerous lesions. Our findings demonstrate that IL-8 levels increase progressively across the entire gastric carcinogenesis spectrum, with effect sizes substantially exceeding those of conventional biomarkers [30]. Given the absence of an established ‘gold standard’ serum biomarker, we benchmarked IL-8 against CEA and CA19-9, the most widely used conventional markers in clinical practice, reflecting standard methodology in biomarker development [12]. IL-8 reflects the underlying inflammatory and tumor microenvironment driven by *H. pylori*-associated carcinogenesis, which may explain its enhanced ability to detect early and progressive disease [12]. Most importantly, we identified three distinct longitudinal IL-8 trajectory patterns that capture the dynamic inflammatory process preceding malignant transformation. The High-Accelerating phenotype, characterized by baseline IL-8 exceeding 52.3 pg/mL and steep acceleration beginning at 12 months, preceded endoscopic cancer detection by a median of 6 months and captured all progressors in our cohort. The progression rate of 9% over 24 months **(**Table 5**)** is consistent with previous cohort studies [23]. As shown in Table 7, both baseline IL-8 and intestinal metaplasia remained independently associated with progression in multivariable analysis. As shown in Table 7, both baseline IL-8 and intestinal metaplasia remained independently associated with progression in multivariable analysis.

In the following thematic sections, we first discuss findings from our baseline cross-sectional comparison of the five study groups, followed by insights from the 24-month longitudinal cohort. We integrate our results with regional and international evidence, explore underlying mechanisms at molecular and cellular levels, and discuss the profound clinical and public health implications of IL-8-based risk stratification.

Our cross-sectional analysis demonstrated a striking progressive increase in serum IL-8 levels across the histological spectrum of gastric carcinogenesis **(Fig. 3**; Table 2**)**, with histopathological confirmation providing a robust foundation for interpretation. Mean IL-8 concentrations rose from 9.7 pg/mL in healthy controls to 42.1 pg/mL in atrophic gastritis, 64.8 pg/mL in intestinal metaplasia, and 107.4 pg/mL in gastric cancer patients, with a monotonic increase confirmed by linear trend analysis [7]. Effect sizes were exceptionally large (Cohen’s d 3.42 and 2.18), substantially exceeding those of conventional biomarkers. These findings suggest that IL-8 elevation begins early in the precancerous cascade and intensifies with disease progression, a hypothesis supported by our longitudinal trajectory analyses [17]. The absence of significant differences in bacterial density across *H. pylori*-positive groups confirms that IL-8 elevation reflects the severity of precancerous changes rather than infection intensity.

As detailed in Table 4, serum IL-8 correlated significantly with all histopathological parameters, most strongly with intestinal metaplasia grade (ρ = 0.64, *p* < 0.001). Among patients with intestinal metaplasia, those with incomplete (type III) subtype had significantly higher IL-8 levels than those with complete (type I) subtype (71.4 ± 12.8 vs. 58.2 ± 11.6 pg/mL, mean difference 13.2 pg/mL, 95% CI: 7.1–19.3, *p* < 0.001), demonstrating that IL-8 reflects not just the presence but the biological aggressiveness of metaplasia. In gastric cancer patients, IL-8 levels correlated significantly with tumor stage **(**Fig. 4; Table 3**)**, with progressive increases from stage I through stage IV [25]. No significant difference was observed between intestinal-type and diffuse-type cancers, indicating that IL-8 elevation is a common feature of gastric malignancy regardless of histological subtype [25]. Our findings align with and substantially extend a growing body of evidence implicating IL-8 in gastric carcinogenesis [17–19]. A nested case-control study within the Shanghai Men’s Health Study by Epplein and colleagues [29] demonstrated that individuals with IL-8 levels above the lowest quartile had approximately two-fold increased odds of gastric cancer, consistent with the progressive elevation we observed. Similarly, a comprehensive meta-analysis by Wang and colleagues [30], pooling eight studies comprising nearly two thousand patients, confirmed that high IL-8 expression was associated with poor prognosis in gastric cancer. Recent mechanistic work by Chang and colleagues [31] has further elucidated that PADI4 promotes epithelial-mesenchymal transition in gastric cancer cells via upregulation of IL-8, while Ma and colleagues [32] established that an IL-8-FAK-IL-8 positive feedback loop operates in gastric cancer cells, promoting proliferation and migration. Studies examining the effects of *H. pylori* eradication on IL-8 expression by Hamaguchi and colleagues [33] and Liao and colleagues [34] have demonstrated that successful eradication significantly reduces mucosal IL-8 levels. Furthermore, dynamic changes in serum IL-8 during immunotherapy have been shown to predict treatment response in advanced gastric cancer by Liu and colleagues [35]. Early work by Shimoyama and colleagues [36] demonstrated that chemokine mRNA expression in gastric mucosa correlates with *H. pylori* cagA positivity and severity of gastritis. Genetic studies have further implicated IL-8 in gastric carcinogenesis, with Li and colleagues [37] demonstrating that IL-8-251 gene polymorphisms increase the risk of atrophic gastritis and intestinal metaplasia in a high-risk Chinese population, providing additional evidence for the role of IL-8-mediated inflammation in the precancerous cascade.

The progressive increase in IL-8 across the carcinogenesis spectrum reflects the evolving inflammatory microenvironment that characterizes each stage of gastric carcinogenesis at the molecular and cellular levels [15]. Experimental studies have demonstrated that *H. pylori* virulence factors, particularly CagA, can induce IL-8 expression through activation of NF-κB [16, 38]. CagA translocates into host cells via the type IV secretion system, leading to NF-κB nuclear translocation. However, we did not assess *H. pylori* virulence determinants, including cagA, cag pathogenicity island integrity, vacA genotype, oipA, babA, and EPIYA motif patterns, and therefore cannot attribute the observed IL-8 elevation specifically to CagA-mediated mechanisms in this cohort. Accordingly, the proposed mechanistic pathway should be interpreted as a biologically plausible explanatory framework rather than direct mechanistic evidence derived from the present cohort.

Studies by Lee and colleagues [39] have demonstrated that the benefit of Helicobacter pylori eradication in reducing gastric cancer risk varies according to patient age, highlighting the importance of timely intervention in the inflammatory cascade. The work of Tu and colleagues [40] demonstrated that overexpression of interleukin-1β induces gastric inflammation and cancer while mobilizing myeloid-derived suppressor cells in mice. As inflammation persists, IL-8 recruits neutrophils to the gastric mucosa through CXCR1 and CXCR2 receptor binding [17, 41]. This neutrophil-tumor cell crosstalk creates a mutagenic microenvironment characterized by reactive oxygen and nitrogen species, which induce DNA damage, including 8-oxoguanine lesions, double-strand breaks, and microsatellite instability [15, 41]. The correlation between IL-8 and activity grade captures this critical mechanistic link between inflammation and genomic damage.

The particularly strong correlation between IL-8 levels and intestinal metaplasia grade suggests that IL-8 may play an active role in metaplastic transformation through convergent mechanisms [36–37]. IL-8 promotes epithelial-mesenchymal transition via PADI4-mediated chromatin remodeling [17, 31] and activates focal adhesion kinase signaling through an IL-8-FAK-IL-8 positive feedback loop that sustains inflammation and promotes cell migration [32]. Additionally, IL-8 may induce epigenetic modifications favoring intestinal differentiation through recruitment of myeloid-derived suppressor cells [38–40]. The stage-dependent increase in IL-8 among gastric cancer patients reflects the role of IL-8 in promoting angiogenesis and metastasis [18–19]. IL-8 upregulates vascular endothelial growth factor expression through HIF-1α-dependent and independent mechanisms [17], explaining the correlation between IL-8 and microvessel density observed in gastric cancer tissues [18]. In advanced stages, tumor-associated neutrophils become a major source of IL-8, creating a feed-forward loop wherein IL-8 recruits neutrophils that themselves produce IL-8, amplifying the inflammatory and angiogenic response [41].

A critical finding of our study is that IL-8 elevation in gastric cancer is independent of *H. pylori* infection status. When stratified by tumor stage as shown in Table 3, there were no significant differences in IL-8 levels between *H. pylori*-positive and *H. pylori*-negative gastric cancer patients at any stage, with all p-values exceeding 0.50 [25]. Furthermore, the progressive increase in IL-8 with advancing stage was observed in both infection-status groups, with p for trend less than 0.001 for both. These findings suggest that once malignant transformation has occurred, IL-8 elevation in established cancer appears more strongly associated with tumor-related inflammatory signaling than infection status [17].

Even more compelling is our analysis of IL-8 dynamics following *H. pylori* eradication, presented in Table 9 and illustrated in Fig. 8. Among successfully eradicated patients, IL-8 levels decreased significantly from baseline to six months, while no significant change was observed in patients with persistent infection. Studies by Hamaguchi and colleagues [33] and Liao and colleagues [34] have similarly demonstrated that *H. pylori* eradication decreases gastric mucosal IL-8 expression. However, when stratified by eventual outcome, a striking pattern emerged: successfully eradicated non-progressors showed a significant early IL-8 decrease, while the eradicated progressors showed no significant early change. Critically, all nine progressors were classified in the High-Accelerating trajectory group regardless of eradication status, indicating that persistent IL-8 elevation despite successful eradication reflects underlying carcinogenic risk rather than active infection. This aligns with recent work by Liu and colleagues [35] showing that dynamic changes in serum IL-8 during PD-1 blockade immunochemotherapy predict response in advanced gastric cancer.

This differential response to eradication provides critical evidence that IL-8 elevation in progressors is not merely a marker of active infection but reflects intrinsic carcinogenic biology. The presence of the High-Accelerating phenotype across all eradication outcome categories suggests that this trajectory pattern reflects underlying carcinogenic biology rather than eradication status or timing of eradication. Importantly, persistent IL-8 elevation despite successful eradication (whether after first-line or second-line) identifies patients at risk, while normalization of IL-8 indicates low risk. These observations support the use of serial IL-8 monitoring as a dynamic risk stratification tool regardless of *H. pylori* eradication history. The fact that IL-8 decreased in non-progressors after successful eradication confirms that infection drives IL-8 in low-risk individuals. Conversely, the persistent elevation in progressors despite documented cure demonstrates that once the carcinogenic process is established, IL-8 production becomes autonomous, a hallmark of the malignant phenotype. These findings position IL-8 not as a passive inflammatory marker but as an active participant in the neoplastic process, with particular value for risk stratification even in patients who have undergone successful eradication therapy. Notably, even after successful eradication, non-progressors maintained significantly higher IL-8 levels (42.8 pg/mL) than never-infected healthy controls (9.7 pg/mL, *p* < 0.001), suggesting that a history of *H. pylori*-associated precancerous lesions leaves a persistent inflammatory imprint. This finding supports the need for separate risk cut-offs for individuals with versus without prior precancerous lesions, as proposed by the reviewer.

The development of targeted therapies, such as CXCR2 inhibitors, may eventually provide chemoprevention options for high-risk individuals. A phase 2 randomized trial by Armstrong and colleagues [42] evaluated the CXCR2 antagonist navarixin in combination with pembrolizumab in patients with advanced solid tumors, demonstrating the feasibility of targeting the IL-8 pathway in cancer therapy. The immune checkpoint inhibitor trials, such as CheckMate 649, reported by Janjigian and colleagues [43], and the insights into IL-8 as a predictor of immunotherapy response from Yuen and colleagues [44], suggest that our findings may have relevance beyond surveillance to treatment selection. The molecular basis for *H. pylori*-dependent IL-8 production involves multiple bacterial and host factors [38]. Canonical NF-κB activation involves IKKβ-mediated phosphorylation and degradation of IκBα, releasing p50-p65 heterodimers for nuclear translocation, as reviewed by Sun [45]. Non-canonical activation involves NIK-mediated processing of p100 to p52, leading to nuclear translocation of p52-RelB complexes [45, 46]. Additionally, the peptidoglycan recognition protein NOD1 detects *H. pylori* peptidoglycan delivered by the same secretion system, activating NF-κB through RIP2 kinase as demonstrated by Viala and colleagues [46]. The resolution of these pathways following eradication explains the rapid IL-8 decrease in non-progressors.

In contrast, the persistent IL-8 elevation in progressors despite successful eradication suggests that in these individuals, IL-8 production has become autonomous, no longer dependent on the inciting infectious trigger [32]. This autonomy likely reflects the establishment of positive autocrine and paracrine signaling loops within the neoplastic microenvironment [32, 47]. Ma and colleagues [32] recently demonstrated that an IL-8-FAK-IL-8 positive feedback loop operates in gastric cancer cells. Beyond autocrine signaling, paracrine interactions with stromal cells contribute to IL-8 autonomy [47]. Cancer-associated fibroblasts in the tumor microenvironment produce IL-8 in response to tumor-derived factors such as transforming growth factor-beta and platelet-derived growth factor, as reviewed by Kalluri [47]. These cancer-associated fibroblast-derived IL-8 acts on cancer cells to promote proliferation and invasion, while also recruiting myeloid-derived suppressor cells that suppress anti-tumor immunity [40].

Epigenetic alterations may also contribute to IL-8 autonomy [39, 48]. The protective effect of *H. pylori* eradication against gastric cancer is age-dependent, underscoring the importance of cumulative inflammatory burden [39]. Gastric cancer risk is associated with an epigenetic field defect characterized by methylation of tumor suppressor genes in non-cancerous mucosa [48]. IL-8 itself contains multiple CpG sites in its promoter region, and acquired hypomethylation could lead to constitutive expression independent of external stimuli in progressors [48, 49].

The persistence of the High-Accelerating trajectory in progressors regardless of eradication status suggests that once a critical threshold of IL-8 dysregulation is reached, the inflammatory process becomes addicted to this cytokine signaling. This concept of inflammatory addiction parallels the well-established phenomenon of oncogene addiction in cancer biology [50]. Just as tumors driven by mutant EGFR or KRAS become dependent on continued signaling from these oncogenes, the inflammatory microenvironment in high-risk patients may become dependent on IL-8-driven signaling cascades. Breaking this addiction may require targeting the IL-8 pathway directly rather than removing the initial inflammatory trigger [42].

The relationship between circulating IL-8 and cancer risk has been explored in other malignancies; Pine and colleagues [51] demonstrated in a prospective study that increased levels of circulating interleukin-6, interleukin-8, and C-reactive protein were associated with risk of lung cancer. Similarly, Ho and colleagues [52] found that circulating soluble cytokine receptors and colorectal cancer risk in the Women’s Health Initiative Observational Study showed significant associations. The methodology for such trajectory analysis has been refined by Nagin and colleagues [53], who developed group-based multi-trajectory modeling approaches. Genetic variants may influence these relationships, as Savage and colleagues [54] reported that variants of the IL8 and IL8RB genes are associated with risk for gastric cardia adenocarcinoma and esophageal squamous cell carcinoma. Meta-analyses by Xue and colleagues [55] have further confirmed that the interleukin-8 -251 promoter polymorphism is associated with gastric cancer risk.

The most novel aspect of our study is the identification of three distinct longitudinal IL-8 trajectory patterns that predict progression to gastric cancer, as illustrated in Fig. 8 and detailed in Table 8. Using group-based trajectory modeling performed blinded to outcome status, we identified a Stable-Low trajectory comprising 42 patients or 45% of the cohort, characterized by consistently low IL-8 levels throughout follow-up with baseline levels of 32 picograms per milliliter and 24-month levels of 35 picograms per milliliter, demonstrating minimal change over time. We identified a Moderate-Rising trajectory comprising 49 patients or 52% of the cohort, characterized by moderately elevated baseline IL-8 levels of 49 picograms per milliliter with a gradual increase over time, reaching 62 picograms per milliliter at 24 months. We identified a High-Accelerating trajectory comprising only nine patients or 10% of the cohort but demonstrating the most clinically significant features, with high baseline IL-8 levels of 58 picograms per milliliter and a striking acceleration beginning at 12 months, with slope before 12 months of 1.1 picograms per milliliter per month increasing dramatically after 12 months to 4.8 picograms per milliliter per month, resulting in 24-month IL-8 levels of 97 picograms per milliliter. Importantly, while baseline IL-8 thresholds (e.g., > 52.3 pg/mL) provide clinically actionable risk stratification for initial decision-making, longitudinal changes in IL-8 within individuals offer additional value by capturing dynamic disease evolution during follow-up. The trajectory analysis **(**Fig. 8; Table 8**)** and the post hoc high-risk phenotype definition (which incorporates both absolute thresholds and ≥ 50% increase from baseline) operationalize this complementary approach. These two strategies, baseline thresholds for initial triage and serial measurements for dynamic refinement, should be viewed as complementary rather than mutually exclusive.

The senescence-associated secretory phenotype represents a critical link between cellular senescence and inflammation [56]. The Stable-Low trajectory reflects individuals whose IL-8 elevation remains driven by H. pylori-induced inflammation without progression to autonomous cytokine production [16]; IL-8 normalizes after eradication because inflammation remains under homeostatic control [33–34]. The Moderate-Rising trajectory may represent persistent low-grade inflammation without the genetic or epigenetic alterations necessary for malignant transformation [56]. The gradual increase in IL-8 over time could reflect cumulative environmental exposures, aging-related changes in immune function, or low-level bacterial persistence despite apparent eradication. Mechanistically, the Moderate-Rising trajectory may represent a state of inflammatory burnout wherein chronic inflammation has induced cellular senescence in some cell populations. Senescent cells acquire a senescence-associated secretory phenotype characterized by production of IL-8, interleukin-6, and other inflammatory mediators [56].

The High-Accelerating trajectory, which captured all progressors, represents a qualitatively different biological state, one in which IL-8 production has become dysregulated and self-perpetuating [32]. The striking acceleration beginning at 12 months suggests a threshold effect, where cumulative inflammatory damage reaches a critical point, triggering positive feedback loops that drive rapid progression [32]. This threshold may represent the point at which IL-8-FAK-IL-8 autocrine loops become established [32], at which epigenetic silencing of negative regulators occurs [48], or at which tumor-associated neutrophils become a significant source of IL-8 [41].

The six-to twelve-month therapeutic window between trajectory acceleration and endoscopic detection corresponds to the time required for an initiated clone to progress to endoscopically visible carcinoma. Importantly, all cancers detected at the 24-month endpoint were early stage (I or II), indicating that interval cancers did not progress to advanced disease undetected during follow-up. This finding supports that the protocol-mandated 12-month surveillance endoscopy effectively minimized interval detection bias and ensured that the observed IL-8 trajectory patterns truly preceded, rather than coincided with, advanced malignant transformation. Mathematical modeling of tumor growth kinetics, as discussed by Friberg and Mattson [57], suggests that a one cubic centimeter tumor containing one billion cells requires approximately thirty doublings from a single transformed cell [57]. At a typical doubling time of sixty to one hundred days for gastric cancer, this corresponds to five to eight years of preclinical growth [57]. However, the accelerated IL-8 rise we observe likely reflects not just tumor growth but also the establishment of a pro-tumorigenic microenvironment that supports progression [41].

The independent association of baseline IL-8 with progression was confirmed in multivariable analysis, with each 10 pg/mL increase conferring a 2.3-fold increased odds of progression after adjusting for histopathological group (adjusted OR 2.31, 95% CI: 1.48–3.61) **(**Table 7**)**. Importantly, the addition of IL-8 to a model containing only histopathological group significantly improved risk prediction, with a net reclassification improvement of 0.42 (*p* < 0.001) and integrated discrimination improvement of 0.18 (*p* < 0.001) **(**Table 7**)**. These metrics confirm that IL-8 provides incremental prognostic value beyond the information obtained from histopathological classification alone, supporting its role as a complementary biomarker in risk stratification.

The molecular events during this window likely include the acquisition of additional genetic alterations that drive metastatic potential. The Cancer Genome Atlas Research Network [58] provided a comprehensive molecular characterization of gastric adenocarcinoma, identifying recurrent mutations in TP53, ARID1A, CDH1, and RHOA, with distinct mutation patterns in intestinal-type versus diffuse-type cancers [58]. The IL-8-driven inflammatory microenvironment may promote genomic instability through reactive oxygen species-mediated DNA damage, selection of p53-mutant clones resistant to apoptosis, and epigenetic silencing of tumor suppressor genes [15, 48].

From a clinical perspective, this therapeutic window has profound implications [42, 59–60]. Potential strategies could include anti-inflammatory agents such as COX-2 inhibitors that have shown chemopreventive effects in gastrointestinal neoplasia, as confirmed by high-quality umbrella reviews [59], potentially by reducing IL-8 production through NF-κB inhibition. For patients with localized high-risk lesions identified through intensive surveillance, endoscopic submucosal dissection could provide curative intervention before progression to invasive cancer, following guidelines established by Ono and colleagues [60].

Collectively, these observations suggest a unifying hypothesis for the High-Accelerating trajectory: chronic *H. pylori* infection, particularly with CagA-positive strains, initiates NF-κB-dependent IL-8 production [16, 38]. In most individuals, eradication therapy removes this trigger, allowing IL-8 levels to normalize and inflammation to resolve, corresponding to the Stable-Low and Moderate-Rising trajectories. However, in a subset of patients, cumulative inflammatory damage and genetic or epigenetic alterations [48] lead to the establishment of self-perpetuating IL-8 signaling loops. IL-8 binding to CXCR1/2 on gastric epithelial cells activates focal adhesion kinase (FAK) [32], creating an autocrine loop that sustains IL-8 production independent of external stimuli. Simultaneously, IL-8 recruits neutrophils and myeloid-derived suppressor cells [40, 41] to the gastric mucosa, where they release reactive oxygen species, causing DNA damage [15] and produce factors that promote angiogenesis [17] and epithelial-mesenchymal transition [31]. The resulting tumor microenvironment, characterized by cancer-associated fibroblasts [47] and sustained inflammation, becomes progressively more permissive for malignant transformation. The acceleration point at 12 months may represent the threshold at which these self-amplifying loops overcome homeostatic controls, driving rapid progression to clinically detectable cancer. This hypothesis, if validated, positions IL-8 not merely as a marker of inflammation but as a central node in a self-sustaining network that may contribute to carcinogenesis through sustained inflammatory signaling once initiated.

The clinical implications of our findings are substantial, particularly in resource-limited settings such as Egypt, where gastric cancer often presents at advanced stages and endoscopic resources are constrained [8–10]. The proposed risk stratification algorithm **(**Table 12**)**, based on baseline and 12-month IL-8 measurements, may offer a framework for personalized surveillance pending validation. Patients classified as low-risk (42% of our cohort) might be considered for extended surveillance intervals in future studies, reducing endoscopic burden and healthcare costs [60]. Conversely, high-risk patients with baseline IL-8 > 52.3 pg/mL and accelerating trajectories may warrant more frequent surveillance and consideration of chemoprevention strategies such as COX-2 inhibitors [59]. The high negative predictive value of baseline IL-8 (98.5%) provides particular clinical utility, as a single low measurement effectively rules out short-term progression risk and may reduce patient anxiety and unnecessary procedures.

The time-dependent analysis further supports a dynamic approach to risk stratification. The optimal IL-8 cut-off increased progressively from 52.3 pg/mL at baseline to 72.8 pg/mL at 18 months, reflecting the rising levels in progressors, while specificity improved from 78.0% to 92.5%, and positive predictive value nearly doubled to 57.1%. These findings suggest that risk stratification may be optimized using time-specific cut-offs, with later measurements providing superior discrimination. The consistently high negative predictive value (98.5–99.0%) across all time points confirms that a single low IL-8 measurement at any point effectively rules out short-term progression risk.

As a pleiotropic chemokine involved in acute and chronic inflammation, IL-8 is not cancer-specific, and elevated levels could theoretically arise from non-malignant inflammatory conditions [17]. However, several lines of evidence support the specificity of our findings for gastric carcinogenesis rather than generic inflammation [29–30]. First, IL-8 has well-established pro-carcinogenic effects, including promotion of angiogenesis, tumor cell proliferation, and epithelial-mesenchymal transition, providing biological plausibility for its role in tumor progression [17–19]. Second, IL-8 levels increased progressively across the histopathological cascade from normal mucosa to gastric cancer, with the strongest correlation specifically with intestinal metaplasia, the direct precancerous lesion [7]. Third, patients with incomplete or type III metaplasia, the higher-risk subtype, had significantly higher IL-8 levels than those with complete metaplasia [7, 37]. Fourth, the stage-dependent increase in IL-8 among cancer patients supports its role as a marker of tumor burden [18]. The strong association between intestinal-type cancers and background intestinal metaplasia (82% vs. 25%, *p* < 0.001) provides robust support for the metaplasia-carcinoma sequence in intestinal-type gastric carcinogenesis. In contrast, the absence of such an association in diffuse-type cancers suggests that these tumors may arise through alternative pathways, potentially involving genetic predisposition or distinct microenvironmental factors. Fifth, our rigorous exclusion of systemic inflammatory conditions, including recent infections, obesity, NSAID use, and metabolic syndrome, minimized confounding from non-gastric inflammation. To minimize misclassification bias, *H. pylori* status was determined using a dual non-invasive testing strategy (stool antigen and UBT) with histopathological confirmation of the 6.4% of cases with initial discordance, consistent with Maastricht VI recommendations [13, 24]. This rigorous approach, combined with post-eradication confirmatory testing, strengthens the internal validity of infection-based subgroup analyses and ensures that IL-8 elevations are attributed to the correct infectious status. Sixth, and most critically, IL-8 elevation persisted after successful *H. pylori* eradication in progressors but decreased significantly in non-progressors, indicating that persistent elevation reflects underlying carcinogenic risk rather than active infection [33–35]. While residual confounding cannot be completely excluded, the predictive accuracy of IL-8 for progression risk remains clinically valuable regardless of mechanistic specificity, analogous to other non-specific but clinically useful biomarkers such as C-reactive protein in cardiovascular risk prediction [17].

A key strength of this study is its prospective longitudinal design with serial IL-8 measurements and protocol-mandated surveillance endoscopy at 12 and 24 months. This design allowed us to examine the temporal relationship between biomarker trajectories and neoplastic transformation, rather than relying on single time-point measurements or cross-sectional comparisons. It demonstrates that IL-8 elevation in progressors persists despite successful *H. pylori* eradication, whereas it normalizes in non-progressors. This differential response confirms that the prognostic value of IL-8 derives from its association with carcinogenic risk rather than active infection, supporting its utility as a surveillance biomarker even in patients who have cleared the infection.

An important distinction emerges from our data: while *H. pylori* infection initiates the inflammatory cascade leading to gastric carcinogenesis [5–7], the subsequent IL-8 elevation in patients who progress reflects tumor biology rather than persistent infection [17]. This is supported by three lines of evidence from our study. First, IL-8 levels were equally elevated in *H. pylori*-positive and *H. pylori*-negative gastric cancers across all stages, as shown in Table 3 [25]. Second, successful eradication led to a significant IL-8 decrease in non-progressors but not in progressors, as detailed in Table 9, consistent with the findings of Hamaguchi and colleagues [33] and Liao and colleagues [34]. Third, the High-Accelerating trajectory phenotype persisted in progressors regardless of eradication status, as illustrated in Fig. 8, aligning with the mechanistic insights from Ma and colleagues [32] on autocrine IL-8 signaling. These findings suggest that IL-8 measurement captures the host’s carcinogenic response independent of active infection, making it valuable as a surveillance biomarker even in patients who have undergone successful eradication therapy [49]. A methodological consideration in biomarker research is the absence of a true ‘gold standard’ comparator for gastric cancer risk stratification. Unlike other malignancies where established serum markers exist, such as alpha-fetoprotein for hepatocellular carcinoma or prostate-specific antigen for prostate cancer, gastric cancer lacks a similarly specific and sensitive biomarker. CEA and CA19-9, despite their widespread use, were never validated for early detection or risk stratification in precancerous populations. Their role in this study, therefore, was not as gold standards but as clinical benchmarks to contextualize IL-8’s performance. The demonstration that IL-8 significantly outperforms these conventional markers (AUC 0.84 vs. 0.62 and 0.58, with net reclassification improvement of 0.42) establishes its incremental value within the current clinical landscape, where no superior alternative exists [12].

The proposed IL-8-based risk stratification algorithm **(**Table 12**)** is intended for patients with established precancerous lesions (atrophic gastritis or intestinal metaplasia) undergoing surveillance, not for population-level screening. It is based on IL-8 measurements alone and does not incorporate other established risk modifiers such as first-degree family history of gastric cancer or detailed histopathological staging (OLGA/OLGIM). Therefore, the algorithm should be viewed as an exploratory tool that complements, rather than replaces, existing surveillance recommendations. Future studies should evaluate whether integrating IL-8 with these established risk factors improves risk prediction and whether the proposed surveillance intervals are safe and cost-effective. Because only nine progression events occurred and the high-risk trajectory comprised only nine patients, the proposed IL-8-based stratification should be interpreted as preliminary and requires validation in larger prospective cohorts with longer follow-up before influencing clinical surveillance strategies.

The primary limitation of this study is the small number of progression events (*n* = 9). Despite using Firth penalized regression and extensive internal validation (bootstrap resampling, 10-fold cross-validation, split-sample validation), model stability remains limited, and findings may be considered exploratory and hypothesis-generating [27, 28]. External validation in larger independent cohorts is required before clinical implementation. Nevertheless, bootstrap validation of the optimal IL-8 cut-off (52.3 pg/mL) demonstrated good stability (95% CI: 48.7–55.9 pg/mL), with 94% of bootstrap samples selecting cut-offs within the optimal range. Second, as a single-country study conducted in Egypt, generalizability to other populations with different genetic backgrounds, *H. pylori* prevalence, and gastric cancer incidence rates remains to be established [8, 9]. IL-8 polymorphisms may vary across populations [54, 55]. Third, IL-8 is a non-specific inflammatory marker. Despite rigorous exclusion of systemic inflammatory conditions and sensitivity analyses for H. pylori eradication, unmeasured confounders (e.g., subclinical infections, dietary factors) cannot be completely excluded [17]. However, the consistency of findings across cross-sectional, longitudinal, and multivariable analyses supports the biological plausibility of IL-8 as a disease-relevant biomarker. Although waterpipe/shisha smoking and several other inflammatory exposures were not prospectively recorded, the potential effect of such unmeasured confounding can be considered quantitatively. The E-value analysis indicated that an unmeasured confounder would need to have relatively strong associations with both elevated baseline IL-8 and subsequent progression, approximately 4.6-fold each, conditional on measured covariates, to move the confidence interval to the null. This analysis does not exclude residual confounding but supports the robustness of the primary association to substantial unmeasured confounding. Although we compared post-eradication IL-8 levels between successfully treated patients and never-infected individuals, the healthy control group did not undergo serial sampling, limiting direct longitudinal comparison.

Fourth, reverse causality in trajectory analysis. Because IL-8 measurements at 6, 12, and 18 months were obtained after enrollment, some progressors may have already developed subclinical malignancy before these measurements. Therefore, the trajectory analysis **(**Fig. 8; Table 8**)** is presented as exploratory and hypothesis-generating. The primary time-to-event analysis using baseline IL-8 (Cox regression, Fig. 7) avoids this concern and provides the main evidence for risk stratification. Fifth, although all baseline biopsies were reviewed to exclude occult malignancy, microscopic foci of cancer at enrollment cannot be entirely ruled out [7]. However, negative 12-month endoscopies in seven of nine progressors mitigate this concern. Sixth, our analysis was restricted to a single biomarker. Combining IL-8 with other inflammatory markers or emerging biomarkers (e.g., microRNAs, circulating tumor DNA) may improve risk stratification and warrants further investigation [49]. Seventh, among the 9 progressors, 7 were intestinal-type, and 2 were diffuse-type. The small number of diffuse-type cases precluded subtype-specific analysis. Given the predominance of intestinal-type cancers (78%), our findings primarily reflect inflammation-driven gastric carcinogenesis associated with *H. pylori*. Applicability to diffuse-type progression requires validation in larger, subtype-specific cohorts.

A methodological limitation of the trajectory analysis **(**Fig. 7; Table 8**)** is potential reverse causality, as IL-8 measurements after enrollment could reflect subclinical malignancy rather than true prediction. To address this, primary time-to-event analysis using only baseline IL-8 demonstrated that baseline IL-8 > 52.3 pg/mL independently predicted progression (adjusted HR: 6.94, *p* < 0.001). The trajectory analysis should therefore be considered exploratory, requiring external validation with even earlier biomarker sampling.

IL-8 is a non-specific inflammatory chemokine that may be influenced by inflammatory conditions unrelated to gastric carcinogenesis. To minimize confounding, we applied predefined exclusion criteria for known inflammatory conditions, including recent acute infections, chronic inflammatory or autoimmune disorders, chronic liver disease, chronic kidney disease, uncontrolled diabetes, and other major inflammatory conditions. However, several inflammatory exposures suggested by the reviewer, including waterpipe/shisha smoking, periodontal disease, latent tuberculosis, parasitic infections, and environmental pollution, were not systematically recorded because they were not included in the original study protocol. We acknowledge that cigarette smoking status is not a surrogate for waterpipe/shisha exposure, and the absence of a statistically significant difference in current smoking cannot be interpreted as equivalence of inflammatory exposure. We agree that unmeasured inflammatory exposures represent potential sources of residual confounding. The concordance of cross-sectional severity, baseline prognostic association, and longitudinal trajectories provides internal consistency that would be difficult to explain by a single unmeasured exposure alone without additional assumptions. Consequently, these variables could not be incorporated into the multivariable analyses and represent potential sources of residual confounding. Given the limited number of progression events, residual confounding from unmeasured inflammatory exposures cannot be fully addressed through statistical adjustment and should be considered when interpreting the findings. Healthy controls were recruited from the same geographic catchment area as the patient groups and were subjected to the same eligibility assessment and predefined exclusion criteria before enrollment. They were selected to represent the source population rather than a deliberately low-inflammatory reference population, thereby helping to minimize the potential for systematic selection bias. Accordingly, the observed associations should be interpreted as supporting the potential prognostic utility of circulating IL-8 within the clinical context studied rather than demonstrating disease-specific biological specificity.

We did not assess *H. pylori* virulence genotypes, including cagA status, cag pathogenicity island integrity, vacA genotype, oipA, babA, or EPIYA motif patterns. Consequently, the present study cannot distinguish whether the observed IL-8 concentrations primarily reflect host inflammatory responses, differences in bacterial virulence, or an interaction between both. The mechanistic discussion of the CagA/NF-κB/IL-8 pathway is therefore presented as biological plausibility supported by the literature rather than cohort-specific evidence. Furthermore, because *H. pylori* strain virulence varies geographically, our findings should be generalized cautiously to populations with different *H. pylori* strain distributions, particularly East Asian populations where CagA-positive and East Asian-type strains are more prevalent and biologically distinct. This should be considered when interpreting the biological and translational implications of our findings.

The additional analyses provided further evidence regarding the relationship between IL-8 and established risk factors. Baseline IL-8 was associated with age, sex, smoking status, and *H. pylori* infection, supporting the importance of accounting for these variables when evaluating its prognostic association. In the exploratory predictive analysis, adding IL-8 to a model incorporating clinical and histopathological risk factors improved discrimination from an AUC of 0.68 to 0.85. Furthermore, IL-8 levels increased progressively across OLGA and OLGIM stages, with a Spearman correlation of 0.61 with OLGIM stage. Addition of OLGA/OLGIM staging did not significantly improve discrimination over IL-8 alone, although this finding should be interpreted cautiously given the limited number of progression events. Collectively, these findings support further investigation of IL-8 as a potential non-invasive adjunctive marker of gastric cancer progression risk rather than as a replacement for established histopathological risk stratification [61–63].

Several key directions for future research emerge from this study. First and foremost, external validation of the trajectory phenotypes and risk stratification algorithm in larger, diverse populations is essential before clinical implementation [28]. International multicenter collaborations should prioritize prospective cohorts with standardized IL-8 measurements and endoscopic surveillance protocols to confirm the reproducibility of the three trajectory classes and the optimal cut-off values identified in our Egyptian cohort, utilizing advanced trajectory modeling methods such as those developed by Nagin and colleagues [53]. Second, the biological mechanisms underlying the High-Accelerating trajectory warrant investigation; studies integrating serial IL-8 measurements with mucosal gene expression profiling, microbiome analysis, and histopathological assessment could elucidate the molecular pathways driving accelerated inflammation and malignant transformation, building on the work of Chang and colleagues [31], Ma and colleagues [32], and the Cancer Genome Atlas Network [58]. Third, the cost-effectiveness of IL-8-guided surveillance compared to the current standard-of-care of regular endoscopic surveillance for all patients with precancerous lesions should be formally evaluated using decision-analytic modeling, incorporating the observed reduction in endoscopic procedures representing 42% of patients classified as low-risk and the potential for earlier cancer detection in high-risk individuals [42]. Fourth, randomized controlled trials are needed to determine whether intervention during the six to twelve month therapeutic window, such as more intensive eradication therapy, anti-inflammatory agents including COX-2 inhibitors which have demonstrated chemopreventive efficacy in gastrointestinal neoplasia [59], CXCR2 antagonists as studied by Armstrong and colleagues [42], or endoscopic resection of high-risk lesions following guidelines such as those of Ono and colleagues [60], can alter the trajectory and prevent progression to invasive cancer [42]. Finally, exploration of multi-marker panels combining IL-8 with other inflammatory cytokines such as interleukin-1 beta and tumor necrosis factor-alpha or emerging biomarkers including microRNAs and circulating tumor DNA may further refine risk stratification and provide mechanistic insights into the host inflammatory response during gastric carcinogenesis, as discussed in comprehensive reviews of IL-8 as a prognostic biomarker [49].

Future studies should also evaluate whether combining IL-8 measurements with detailed anatomical staging of atrophy and intestinal metaplasia (e.g., OLGA/OLGIM) improves risk stratification compared with either modality alone, as the proposed algorithm currently does not incorporate lesion extent. Future studies should also evaluate whether combining IL-8 measurements with established risk factors (e.g., family history, OLGA/OLGIM staging) improves risk stratification and whether the proposed surveillance intervals (e.g., extended intervals for low-risk patients) are safe in prospective validation cohorts.

## Conclusion

This prospective multicenter cohort study demonstrates that baseline serum IL-8 > 52.3 pg/mL is independently associated with an increased hazard of malignant progression (adjusted HR: 6.94, *p* < 0.001). Exploratory trajectory analysis identified a High-Accelerating phenotype that preceded endoscopic cancer detection by a median of 6 months; however, this finding requires external validation and should be interpreted with caution due to potential reverse causality.

IL-8 elevation was independent of *H. pylori* infection status. Successful eradication reduced IL-8 in non-progressors but not in progressors, indicating that persistent elevation reflects carcinogenic risk rather than active infection. The proposed risk stratification algorithm, based on baseline and 12-month IL-8 measurements, identified 42% of patients as low-risk (0% progression) in this cohort, suggesting that extended surveillance intervals may warrant evaluation in future studies rather than immediate clinical adoption, while high-risk patients may benefit from intensified monitoring. Baseline IL-8 > 52.3 pg/mL demonstrated a high negative predictive value (98.5%) for ruling out short-term progression.

These findings support the potential prognostic relevance of IL-8 for gastric cancer risk stratification within this study population, but the results should be interpreted as hypothesis-generating. External validation in independent cohorts with longer follow-up and systematic assessment of inflammatory exposures is required before clinical implementation, particularly in resource-limited settings where endoscopic resources are constrained.

## Data Availability

All summarized findings are included in this published article.
